# *In situ*-activated engineered bacteria release combined dual-therapeutic proteins for tumor immunotherapy

**DOI:** 10.1016/j.isci.2026.118090

**Published:** 2026-11-02

**Authors:** Xin-Ge Wang, Yue-Lan Huang, Xuan-Ren Shi, Zhen-Ping Zou, Ying Zhou, Bang-Ce Ye

**Affiliations:** 1 Laboratory of Biosystems and Microanalysis, State Key Laboratory of Bioreactor Engineering, East China University of Science and Technology, Shanghai 200237, China

**Keywords:** engineered bacteria, closed-loop treatment, synergistic, tumor immunotherapy

## Abstract

The combination of antitumor agents is pivotal for effective tumor immunotherapy. However, achieving precise, *in situ* co-delivery of therapeutic payloads remains a considerable challenge. Here, we developed a dual-payload-loaded *Salmonella typhimurium* strain capable of releasing granulocyte-macrophage colony-stimulating factor (GM-CSF) and CD47 nanobody (CD47nb) in response to tumor hypoxia and bacterial quorum sensing (QS). GM-CSF promotes dendritic cell (DC) maturation and enhances M1-like macrophage infiltration, facilitating antigen presentation and CD8^+^ T cells activation. CD47nb blocks the CD47-SIRPα signaling axis, thereby enhancing macrophage-mediated tumor cells phagocytosis. The combined delivery increased mature DC infiltration from 13.5% to 34.9%, promoted M1-like macrophage activation and CD8^+^ T cell accumulation in the tumor microenvironment (TME), suppressed tumor growth by 83.7%, and extended the median survival from 14 to 28 days compared with PBS-treated mice. In summary, our findings support precise, *in situ* delivery of complementary immunomodulators as a promising strategy for tumor immunotherapy.

## Highlights

• Dual-payload-loaded *S. typhimurium* enables tumor therapy via hypoxia and quorum sensing
• GM-CSF and CD47nb coordinately enhance dendritic cells, macrophages, and T cell activation
• *In situ* activated engineered *S. typhimurium* suppresses tumor growth and extends survival

## Graphical abstract

**Figure:**
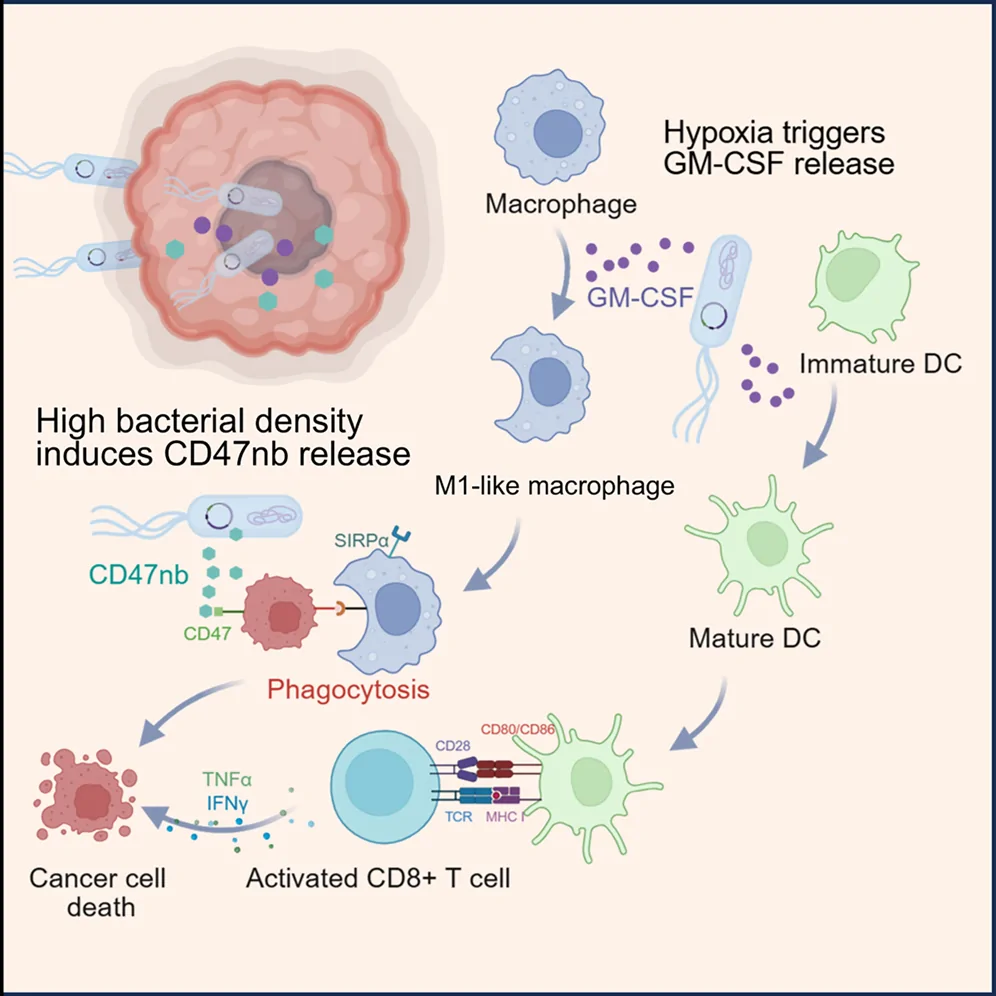

## Teaser

Health sciences; Immunology; Microbiology; Cancer

## Introduction

Tumor immunotherapy, which aims to harness and enhance the host immune system to eliminate cancer cells, has achieved significant advances over the past decade.^1^ Critical to this strategy are antigen-presenting cells (APCs), particularly dendritic cells (DCs) and macrophages, which orchestrate T cell activation and mediate tumor clearance.^2^^,^^3^ However, tumor microenvironment (TME) poses formidable barriers to effective immune responses. Owing to adverse conditions such as hypoxia, nutrient deprivation, and acidosis,^4^^,^^5^^,^^6^^,^^7^ the majority of tumor-associated macrophages adopt a pro-tumoral M2 phenotype rather than an anti-tumor M1 state.^8^ Moreover, the immunosuppressive TME impairs DC maturation, thereby limiting T cell priming and undermining systemic antitumor immunity.^3^ Compounding these challenges, cancer cells exploit intrinsic molecular mechanisms to evade immune surveillance. CD47, overexpressed on the surface of cancer cells, is a ligand that engages signal regulatory protein α (SIRPα) on macrophages to deliver a “do not eat me” signal, thereby inhibiting phagocytosis.^9^^,^^10^ In this way, monotherapy with a single immunomodulatory agent often fails to elicit efficient responses.^11^^,^^12^^,^^13^ Moreover, conventional non-targeted delivery platforms often suffer from insufficient local concentrations of therapeutic agents, compromising treatment efficacy and increasing the risk of off-target toxicities.^14^ In light of these restrictive factors, *in situ* delivery of coordinated therapeutic agents is urgently required to rebuild the TME and boost the anti-tumoral immunotherapy.^15^

Owing to their intrinsic immunogenicity and natural tropism for the hypoxic necrotic cores of tumors,^16^ bacteria have emerged as promising vectors for localized delivery of diverse therapeutic payloads, including cytotoxic proteins,^17^^,^^18^^,^^19^^,^^20^ cytokines,^21^^,^^22^ nanobodies,^23^^,^^24^ and small interfering RNAs.^25^ Among these, attenuated *Salmonella typhimurium* (*S. typhimurium*) has attracted particular interest in bacterial cancer therapy.^26^ As a facultative anaerobe accumulating in tumor tissues about 10,000 times that of healthy organs,^27^ *S. typhimurium* is well suited for engineering as an *in situ* delivery vehicle for therapeutic proteins at the tumor site in a bacterial density-dependent manner.^28^ Moreover, incorporating sensor elements responsive to markers at the disease site into engineered bacteria enables coordinated delivery of multiple therapeutic proteins with precise spatiotemporal control to significantly improve both safety and efficacy of the treatment.^29^

Here, we engineered *S. typhimurium* VNP20009,^30^ termed VNPΔ-CGA, to act as a dual-payload delivery system that couples release signals with immune-modulating functions. In the hypoxic TME, VNPΔ-CGA secretes granulocyte-macrophage colony-stimulating factor (GM-CSF), an immune-stimulating cytokine that promotes DC maturation and enhances macrophage polarization toward a pro-inflammatory M1-like phenotype,^31^^,^^32^^,^^33^^,^^34^ thereby enhancing antigen presentation and subsequent CD8^+^ T cell activation. As bacterial proliferation proceeds, quorum sensing (QS) is activated, triggering expression and release of a CD47 nanobody (CD47nb), an immune checkpoint blocker targeting the CD47-SIRPα axis and strengthen macrophage-mediated tumor clearance and antigen presentation.^23^ By linking payload release to the bacterial environmental cues, hypoxia for GM-CSF and QS for CD47nb, the system enables spatiotemporally coordinated immune stimulation. The combined activities of GM-CSF and CD47nb potentiate the antitumor efficacy of the engineered bacteria. Together, this study presents an engineered bacteria-based strategy for spatiotemporally coordinated delivery of complementary immunomodulatory proteins within the TME, providing a potential approach for improving tumor immunotherapy.

## Results

### Engineered *S. typhimurium* for response to TME biomarkers

To verify the effectiveness of GM-CSF and CD47nb, we purified these two proteins and verified their functions *in vitro* using the experimental methods shown in Figures S1A and S1B. Briefly, purified GM-CSF was incubated with RAW264.7 cells, and the changes in the proportion of CD80^+^ CD86^+^ RAW264.7 cells were subsequently measured. It was observed that the proportion of CD80^+^ CD86^+^ RAW264.7 cells in the GM-CSF group increased to 17.6%, compared with 0.1% in the blank group (Figures S1C and S1D). Similarly, when gradient-diluted CD47nb was co-incubated with mouse colorectal cancer cells (CT26), we found that the proportion of CD47-positive cells decreased as the concentration of CD47nb increased, indicating that CD47nb was able to bind to CD47 on the surface of tumor cells (Figure S1E).

Next, we explored whether engineered bacteria could regulate therapeutic protein release in response to the hypoxic TME and high bacterial density. Three oxygen-dependent promoters were tested in *S. typhimurium* according to the design shown in Figure 1A.^35^^,^^36^ As shown in Figure 1B, under anaerobic conditions, the yfiD-m-Lux strain had the highest luminescence output with a 103.8-fold induction. Subsequently, we implemented the QS system and put the *sfGFP* reporter gene downstream of the p_lux_ promoter. This QS system consists of the autoinducer synthase LuxI, the transcriptional regulator LuxR, and the p_lux_ promoter. LuxI synthesizes N-(3-oxohexanoyl)-L-homoserine lactone (3OC6HSL), which activates LuxR to initiate transcription downstream of p_lux_ (Figure 1C).^28^ Our results demonstrated a clear positive correlation between fluorescence intensity and bacterial cell density, confirming the density-dependent activation of the QS circuit (Figures 1D and 1E). Based on the preceding validation, we proceeded to assemble functional modules for the controlled secretion of therapeutic proteins. We selected the high-performing yfiD-m promoter to trigger HA-tagged GM-CSF expression under anaerobic conditions, with the peptide MISSSSIS^37^ facilitating its secretion (Figure 1F). Western blot (WB) results confirmed GM-CSF secretion into the medium by MISSSSIS (Figure 1G). We harnessed the QS system to regulate the expression of Flag-tagged CD47nb and the secretory peptide OmpA to mediate its release from bacteria (Figure 1H). The engineered bacteria were capable of both expressing and secreting CD47nb, unlike wild-type *S. typhimurium* (Figure 1I). Collectively, these results demonstrated that the release of two distinct therapeutic payloads from our engineered bacterial chassis could be effectively regulated by two key physiological stimuli: the hypoxic TME and bacterial population density.

**Figure 1:**
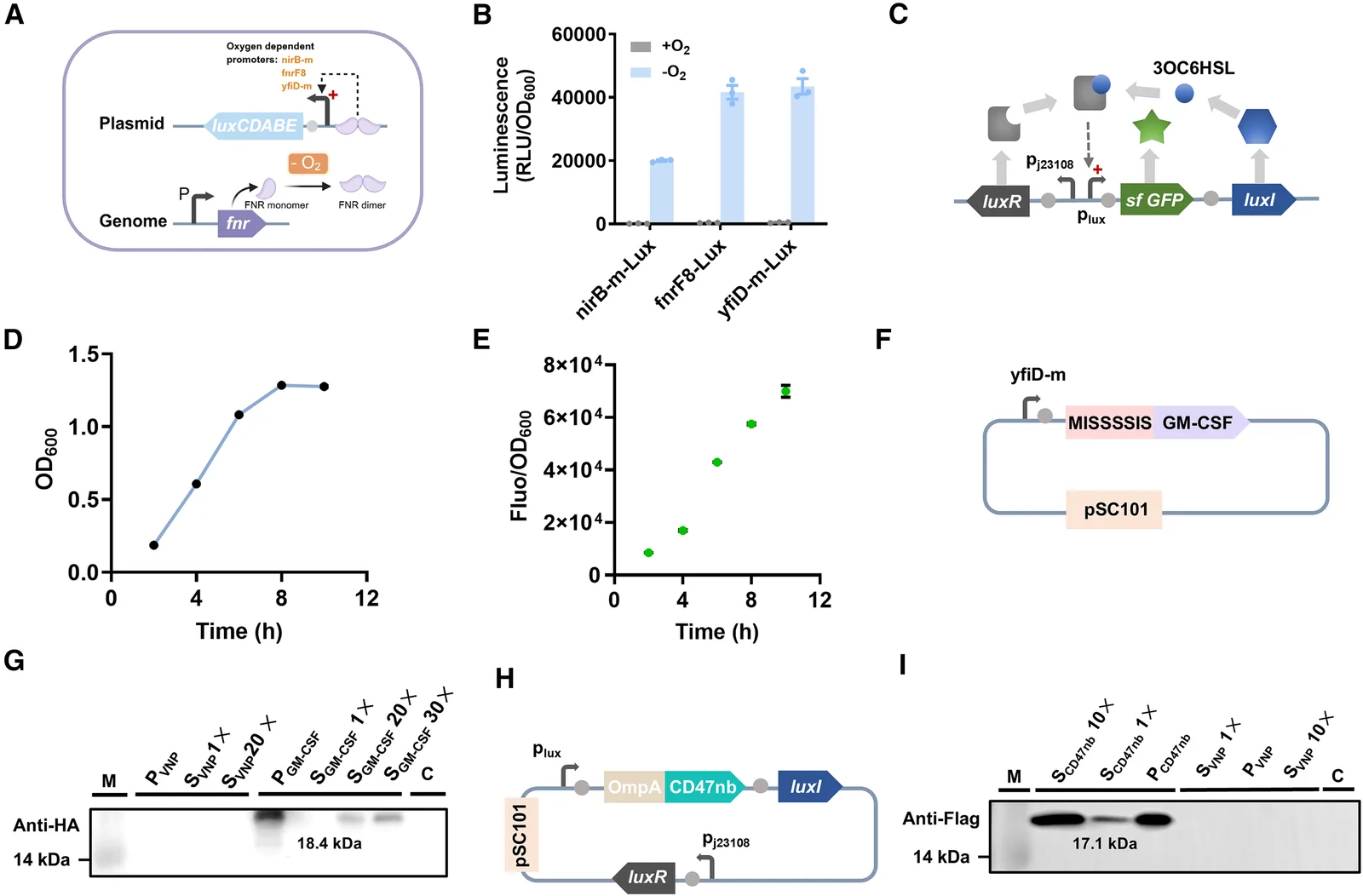
Gene circuit design of QS system regulated CD47nb release or anaerobic environment induced GM-CSF production (A) Schematic diagram of hypoxia induction and anaerobic promoter screening. (B) Quantification of luminescent intensities of bacteria nirB-m-Lux, fnrF8-Lux, yfiD-m-Lux after aerobic and anaerobic incubation for 8 h (mean ± SEM, *n* = 3). (C) Schematic diagram of bacterial lux quorum sensing (QS) system. (D and E) Verification of the lux QS system through the sf GFP reporter gene (mean ± SEM, *n* = 3). (F) Schematic of the engineered plasmid within HA-tagged GM-CSF expression and secretion under the control of yfiD-m promoter and MISSSSIS peptide, respectively. (G) GM-CSF expression and secretion analysis through western blot of pellets and supernatants of VNP20009 and VNP-MIS-G culture solutions. M, marker; C, loading control; P, pellet; S, supernatant; 20×, 20-fold concentrated supernatant; 30×, 30-fold concentrated supernatant. (H) Schematic of the engineered plasmid with Flag-tagged CD47nb expression under the control of the lux QS system and secretion mediated by the OmpA peptide. (I) CD47nb expression and secretion analysis through western blot of pellets and supernatants of VNP20009 and VNP-C culture solutions. M, marker; C, loading control; P, pellet; S, supernatant; 10×, 10-fold concentrated supernatant.

### Construction and characterization of a dual-payload-loaded *S. typhimurium* strain

We further developed an engineered bacterium capable of simultaneously and autonomously regulating the release of GM-CSF (induced by hypoxic conditions) and CD47nb (induced by QS signals) (Figure 3A). To maximize the functional output of this system, we optimized the secretion module based on a previously reported signal peptide and InfB tag optimization strategy for bacterial cytokine delivery. Specifically, we selected the outer membrane protein-derived OmpC and OmpF signal peptide sequences and evaluated the effect of placing the solubility-enhancing tag InfB at either the N terminus or C terminus of GM-CSF (Figure 2A; Table S1).^38^^,^^39^ Concurrently, we monitored the production of CD47nb. The concentrations of CD47nb (Figure 2B) and GM-CSF (Figure 2C) in the culture supernatant were determined by ELISA. The results demonstrated that the circuit combining the OmpF secretion peptide with an N-terminal InfB tag (OmpF-infB-GM-CSF) produced the highest extracellular levels of both GM-CSF and CD47nb. This optimized dual-function strain was designated VNP-CG for subsequent experiments.

**Figure 2:**
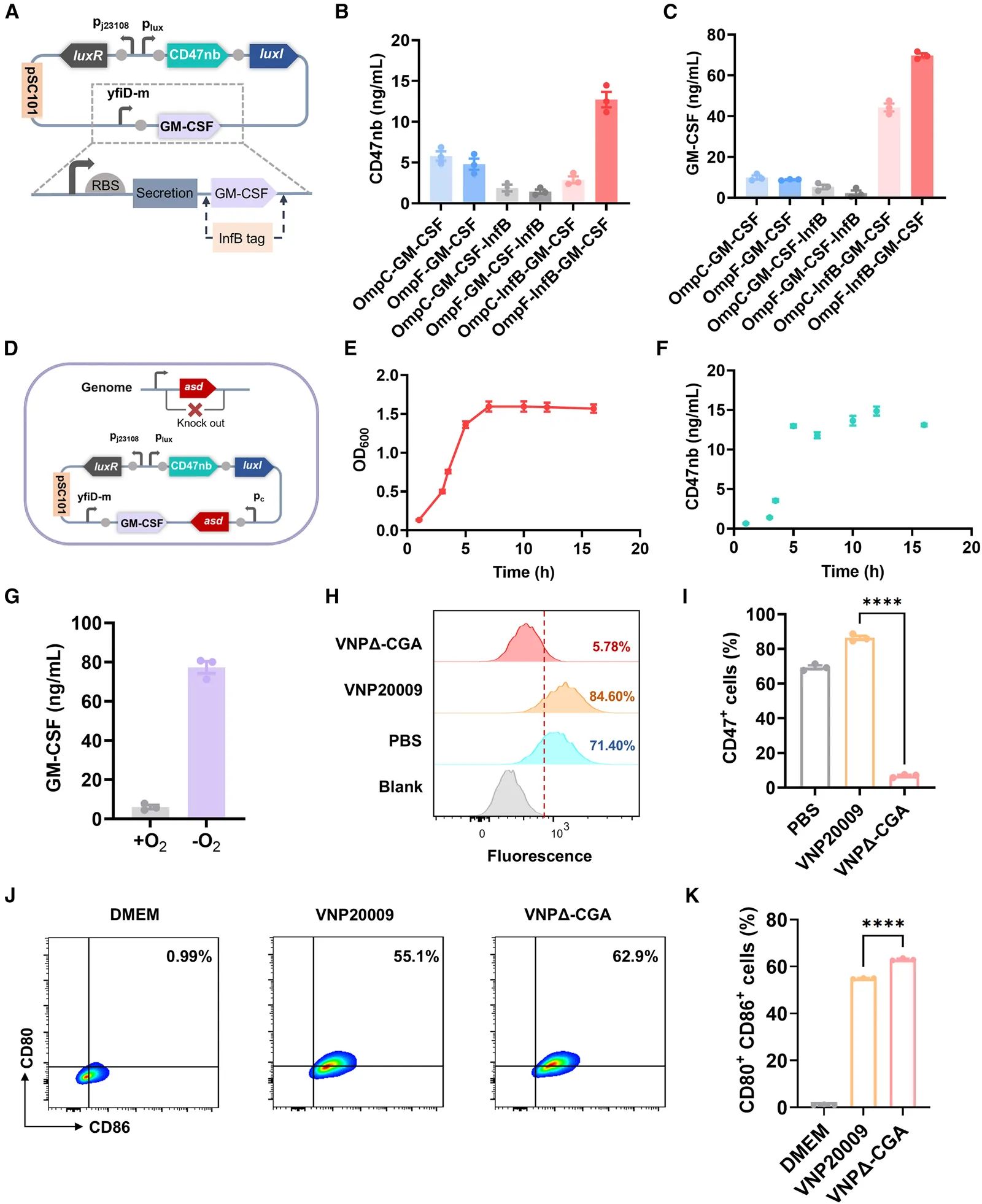
Optimization of the engineered plasmid in VNP20009 for secretion of bioactive CD47nb and GM-CSF (A) Regarding the design for optimizing the secretion of GM-CSF after the integration of the genetic circuits. (B) Comparison of CD47nb concentrations in bacterial culture supernatants among six optimized strains after 12 h culturing (mean ± SEM, *n* = 3). (C) Comparison of GM-CSF concentrations in bacterial culture supernatants among six optimized strains after 12 h (mean ± SEM, *n* = 3). (D) The diagram of the engineered VNP20009 stably expressing GM-CSF and CD47nb. (E) Bacterial growth curve of VNPΔ-CGA within 16 h (mean ± SEM, *n* = 3). (F) Concentration change curve of CD47nb in VNPΔ-CGA culture supernatant within 16 h (mean ± SEM, *n* = 3). (G) Quantification of GM-CSF secretion of VNPΔ-CGA cultured in anaerobic environment for 8 h (mean ± SEM, *n* = 3). (H) Flow cytometry analysis of CD47 blocking after 24 h of incubation between CT26 cells and VNPΔ-CGA culture supernatants. (I) The proportion of CD47^+^ cells after 24 h of incubation. (mean ± SEM, *n* = 3). *p* values were analyzed by one-way ANOVA, ∗∗∗∗*p* ≤ 0.0001. (J) Flow cytometry analysis of M1-like macrophage activation after 48 h incubation between RAW264.7 and 2-fold diluted bacterial culture supernatants. (K) The proportion of CD80^+^ CD86^+^ cells after 48 h of incubation. (mean ± SEM, *n* = 3). *p* values were analyzed by one-way ANOVA, ∗∗∗∗*p* ≤ 0.0001.

To construct a therapeutic bacterial strain with guaranteed genetic stability, we established a balanced-lethal host-vector system based on the *asd* gene (encoding aspartate semialdehyde dehydrogenase).^40^ The chromosomal *asd* gene was deleted in the *S. typhimurium* chassis, creating an auxotrophic host (VNPΔ*asd*). A constitutively expressed *asd* cassette was then inserted into the therapeutic plasmid, making plasmid carriage essential for survival. The obtained strain was named VNPΔ-CGA (Figure 2D). This stabilization system did not compromise bacterial growth (Figure S2A). Its effectiveness was demonstrated through serial passage in antibiotic-free medium. For the control strain VNP-CG, plasmid loss initiated early during subculturing. By the third generation, a subpopulation of plasmid-free cells emerged, indicating the onset of instability. By the ninth generation, only approximately 27% of the VNP-CG population retained the engineered plasmid, while VNPΔ-CGA maintained the plasmid in virtually 100% (Figure S2B). Functionally, the secretion of the CD47nb by VNPΔ-CGA was growth-associated. The concentration in the supernatant plateaued after the bacterial density reached stationary phase, with a maximum level of 14.8 ng/mL (Figures 2E and 2F). After anaerobic cultivation for 8 h, the ELISA assay showed that the titer of GM-CSF in the supernatant of VNPΔ-CGA culture was 77.3 ng/mL (Figure 2G).

To assess the biological activity of CD47nb and GM-CSF produced by engineered bacteria, we co-incubated PBS, wild-type *S. typhimurium* VNP20009 culture supernatant, and engineered bacteria culture supernatant with CT26 cells for 24 h. After incubation, we measured the CD47-positive cell percentage. In the engineered bacteria group, this rate dropped to 6.83%, significantly lower than the other two groups (Figures 2H and 2I), indicating that CD47nb effectively bound to CD47 protein, blocking the CD47-SIRPα signaling pathway. Similarly, wild-type *S. typhimurium* and engineered bacteria were cultured anaerobically for 12 h, and RAW264.7 cells were co-incubated with DMEM, diluted wild-type *S. typhimurium* supernatant, or engineered bacteria supernatant for 48 h. Flow cytometry analysis showed CD80^+^ CD86^+^ cells at 0.99%, 55.1%, and 62.9%, respectively (Figures 2J and 2K). The proportion of CD80^+^ CD86^+^ cells in the engineered bacteria group was 7.8 percentage points higher than in the negative control group, suggesting that GM-CSF expression can promote M1-like activation of RAW264.7 cells.

### Anti-tumor effect of VNP-CG and VNPΔ-CGA

To investigate the impact of plasmid stability on the therapeutic performance of engineered bacteria, we first examined the *in vivo* maintenance of the recombinant plasmid in VNPΔ-CGA. Following intravenous (i.v.) administration into BALB/c mice bearing subcutaneous (s.c.) CT26 tumors, tumors were harvested 24 h post-injection, homogenized, and plated onto LB agar containing streptomycin (Sm) alone or both streptomycin and kanamycin (Sm + Kan). Bacterial growth analysis revealed no significant difference in colony counts of VNPΔ-CGA on Sm versus Sm + Kan plates, indicating stable plasmid maintenance *in vivo*. In contrast, VNP-CG colonies on Sm + Kan plates were reduced by approximately four orders of magnitude compared with those on Sm plates, demonstrating substantial loss of the engineered plasmid (Figures 3A and 3B). By introducing the *luxCDABE* reporter cassette into the engineered plasmid, IVIS imaging of the dissected tumors also confirmed the retention of the plasmid in tumor-colonizing engineered bacteria (Figure 3C).

**Figure 3:**
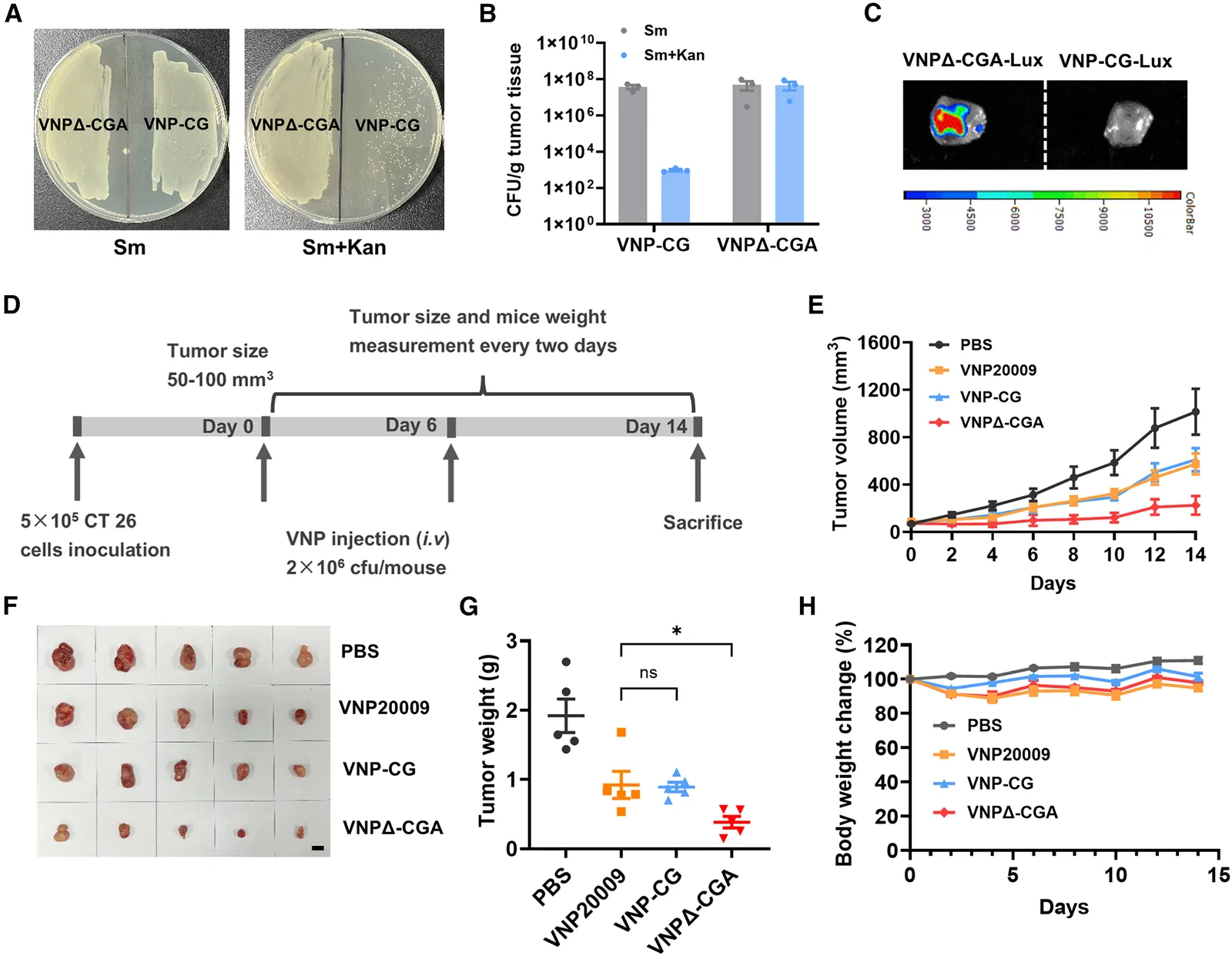
VNPΔ-CGA suppressed tumor growth more effectively with stable expression of therapeutic proteins (A) Bacteria growth on Sm and Sm + Kan agar plates after 24 h of bacterial injection (*KanR* was inserted into the engineered plasmid). (B) Quantification of bacterial colonization of VNP-CG and VNPΔ-CGA with or without functional engineered plasmids in tumors after 24 h of bacterial injection (mean ± SEM, *n* = 3). (C) Visualization of engineered plasmid retention in tumor-colonizing VNP-CG and VNPΔ-CGA by bioluminescence imaging of excised tumors 24 h after injection. (D) Therapeutic schedule of CT26 subcutaneous tumor-bearing mice. (E) Average tumor growth curves of different treatment groups within 14 days (mean ± SEM, *n* = 5). Statistical analysis was performed using two-way repeated-measures ANOVA followed by Sidak’s multiple comparisons test. (F) Tumor sizes of different treatment groups at the 14th day (scale bars, 10 mm). (G) Tumor weights of different treatment groups at the 14th day (mean ± SEM, *n* = 5), *p* values were analyzed by one-way ANOVA, ∗*p* ≤ 0.05. (H) Mice body weight changes of different treatment groups (mean ± SEM, *n* = 5).

We then assessed the antitumor efficacy of VNP-CG and VNPΔ-CGA in a CT26 tumor-bearing mouse model (Figure 3D). VNPΔ-CGA exhibited superior antitumor activity compared with the wild-type *S*. *typhimurium* strain VNP20009 (Figures 3E–3G). Tumor sizes in the VNP-CG group were comparable to those in the VNP20009 group (Figure 3E). At the endpoint, the average tumor weights were 1.838, 0.922, 0.881, and 0.311 g for PBS, VNP20009, VNP-CG, and VNPΔ-CGA groups, respectively, with tumors in the VNPΔ-CGA group weighing approximately one-sixth of those in the PBS group and one-third of those in the VNP20009 group. VNP-CG did not exhibit improved therapeutic efficacy compared with VNP20009, as tumor growth and tumor weight were comparable between the two groups. In contrast, VNPΔ-CGA significantly reduced tumor weight compared with VNP20009 (Figure 3G). These results indicated that stable plasmid maintenance is indispensable for realizing the antitumor potential of engineered bacteria.

### Evaluation of the anti-tumor effects of *S. typhimurium* loaded with dual and single therapeutic proteins

To evaluate the anti-tumor effects of *S. typhimurium* engineered to deliver dual or single therapeutic proteins, we constructed strains VNPΔ-CA and VNPΔ-GA (Figures S3A and S3B). In VNPΔ-CA, expression of CD47nb is governed by the QS system (Figure S3C), whereas GM-CSF production in VNPΔ-GA is regulated by hypoxia-responsive elements (Figure S3D). Seven days after s.c. inoculation of CT26 cells into BALB/c mice, once tumors reached 50–100 mm^3^, the mice were randomized into five groups: PBS (control), *S. typhimurium* VNP20009, VNPΔ-CA (QS-regulated CD47nb), VNPΔ-GA (hypoxia-regulated GM-CSF), and VNPΔ-CGA (both CD47nb and GM-CSF regulation). Except for the PBS group, mice received 2 × 10^6^ CFU (colony-forming unit) of bacteria via tail vein injection (Figure 4A). Three days post-administration, tumor tissues were harvested for WB and ELISA analyses to verify *in situ* expression of CD47nb and GM-CSF (Figures 4B and 4C). The data suggested that the engineered strains were activated by the intratumoral (i.t.) hypoxic microenvironment and QS signaling, resulting in localized release of the therapeutic proteins. Tumor volume and body weight were monitored every two days until day 12 (Figures 4D–4I). Compared with PBS treatment, all bacterial treatment groups showed reduced tumor growth, with engineered bacterial groups exhibiting more effective tumor suppression (Figure 4D). At the endpoint, tumor weight analysis further confirmed the superior efficacy of VNPΔ-CGA (Figure 4J). VNPΔ-CGA treatment resulted in significantly reduced tumor weight compared with VNP20009 treatment, whereas VNPΔ-CA and VNPΔ-GA showed no significant difference compared with VNP20009. Survival analysis further demonstrated the therapeutic benefit of VNPΔ-CGA. Survival curves showed that VNPΔ-CGA significantly prolonged survival compared with the PBS group (*p* < 0.0001), with a median survival time of 28 days versus 14 days for PBS. Among all treatment groups, VNPΔ-CGA achieved the longest median survival, compared with 24 days for VNP20009, VNPΔ-CA, and VNPΔ-GA (Figure 4K). Notably, 25% (2/8) of mice in the VNPΔ-CGA group remained alive at the end of the 36-day observation period (Figures S4A–S4E). The change in mouse body weight remained within ethically acceptable limits (˂ 20% loss) (Figure S5). To further validate the antitumor efficacy of VNPΔ-CGA, an MC38 tumor model was established. VNPΔ-CGA treatment inhibited tumor growth and reduced tumor weight compared with PBS treatment, while maintaining stable body weight during the treatment period (Figure S6).

**Figure 4:**
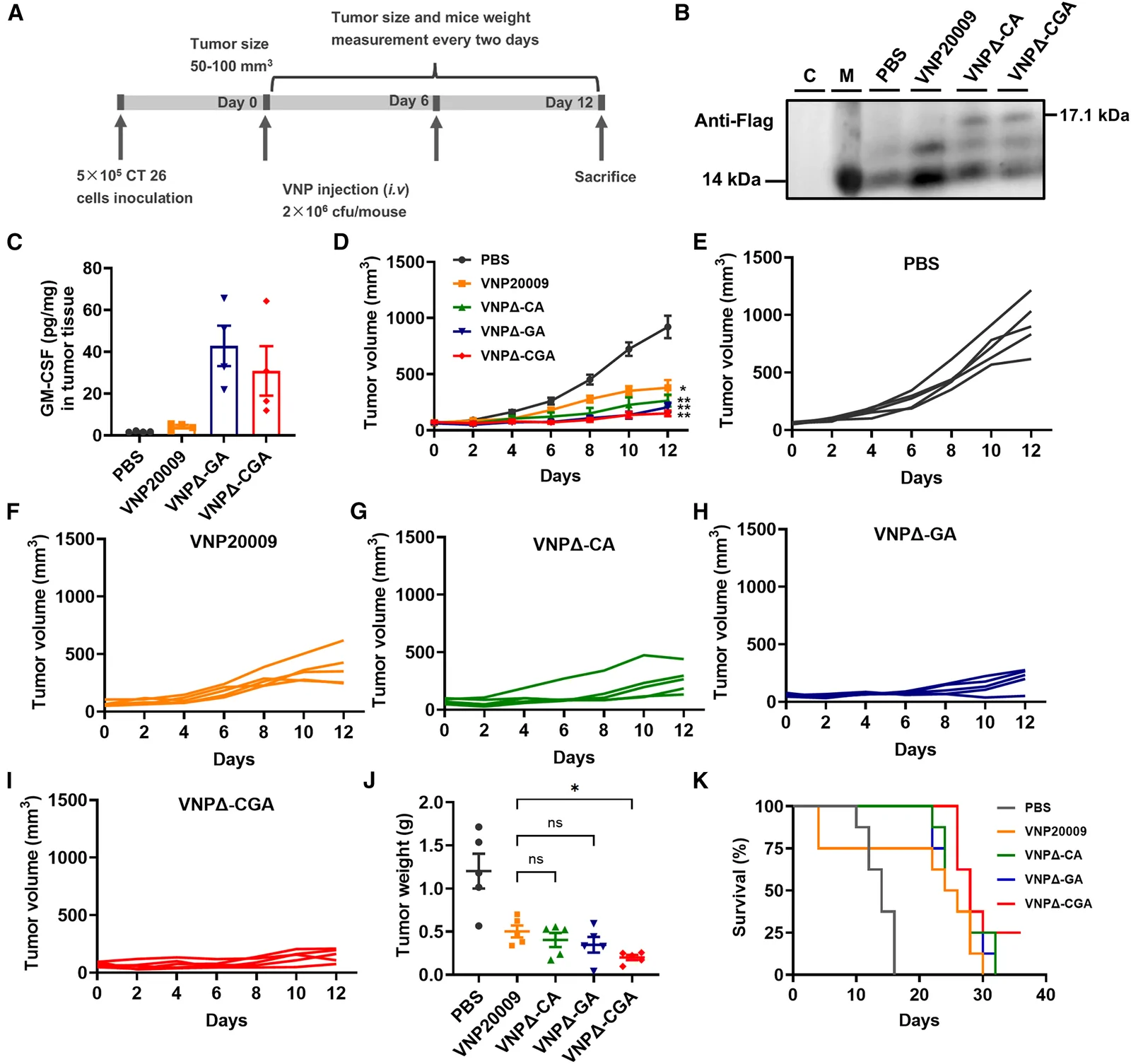
Therapeutic efficacy of VNPΔ-CGA in the CT26 subcutaneous tumor model (A) Therapeutic schedule of CT26 subcutaneous tumor-bearing mice. (B) Western blot analysis of CD47nb producing by engineered VNP20009 in tumors after 72 h of bacterial injection. (C) Quantification of intratumoral GM-CSF between different treatment groups after 72 h of bacterial injection (mean ± SEM, *n* = 4). (D) Average tumor growth curves of different treatment groups within 12 days (mean ± SEM, *n* = 5). *p* values were analyzed by two-way repeated-measures ANOVA followed by Sidak’s multiple comparisons test, ∗*p* ≤ 0.05, ∗∗*p* ≤ 0.01. (E–I) Tumor volume changes of every single mouse in different treatment groups. (J) Tumor weights of different treatment groups at the 12th day (mean ± SEM, *n* = 5). *p* values were analyzed by one-way ANOVA, ns *p* > 0.05, ∗*p* ≤ 0.05. (K) Survival curves of mice in different treatment groups (*n* = 8). Statistical analysis was performed using the Kaplan-Meier method with log-rank test.

Despite the fact that body weight loss in mice treated with VNPΔ-CA, VNPΔ-GA, and VNPΔ-CGA was comparable to that observed in the VNP20009 group, we further evaluated bacterial biodistribution at both an early time point (day 3) and the endpoint of therapy (day 12). At day 3 after administration, all bacterial strains exhibited efficient tumor colonization, with comparable bacterial burdens detected in tumor tissues. At the endpoint of therapy, quantification of residual bacteria in tumor tissues and major healthy organs (spleen and liver) revealed that colonization levels in tumors were similar across all four groups, averaging approximately 8 × 10^8^ CFU/g of tissue. In contrast, our engineered strains displayed markedly lower persistence in healthy organs than VNP20009 following treatment (Figure S7). To further evaluate the systemic safety of bacterial treatment, serum pro-inflammatory cytokines and liver function-related markers were measured at both early post-administration stages and the experimental endpoint. No significant elevation of inflammatory cytokines or hepatic injury-related markers was observed in VNPΔ-CGA-treated mice compared with control groups (Figures S8 and S9), indicating that the engineered bacterial strain did not induce detectable systemic toxicity during treatment.

### Analysis of the antitumor mechanism of dual-payload-loaded engineered *S. typhimurium*

To elucidate the immune response elicited by bacterial therapy, we analyzed immune cell populations in the TME of s.c. CT26 tumor-bearing mice following treatment with engineered *S*. *typhimurium*, focusing on APCs including macrophages and DCs within the TME. We found that the percentages of immune-activated M1-like macrophages (F4/80^+^ CD80^+^) among CD45^+^ cells were as follows: 13.0% in PBS-treated mice, 11.9% in VNP20009, 10.7% in VNPΔ-CA, 18.1% in VNPΔ-GA, and 20.4% in VNPΔ-CGA. Notably, the VNPΔ-GA and VNPΔ-CGA groups showed a pronounced increase in the proportion M1-like macrophages (Figures 5A and 5B). Moreover, the VNPΔ-CA group exhibited the lowest proportion of immunosuppressive M2-like macrophages (CD206^+^) at 5.4%, while the proportions in VNPΔ-CGA, VNPΔ-GA, and VNP20009 were 10.6%, 12.4%, and 12.5%, respectively, and 19.8% for the control group (Figures 5C and 5D). These data indicated that GM-CSF could enhance M1-like macrophage recruitment, whereas CD47nb could attenuate M2-like macrophage infiltration within the TME. Analysis of DCs showed that GM-CSF promoted DC maturation, characterized by high CD80 and CD86 expression. The proportions of mature DCs were 12.1% in PBS-treated mice, 13.5% in VNP20009, 17.8% in VNPΔ-CA, 21.3% in VNPΔ-GA, and 34.9% in VNPΔ-CGA, with the greatest enhancement observed in the dual-regulated VNPΔ-CGA group (Figures 5E and 5F). Correspondingly, the ratios of M1-like to M2-like macrophages were 0.7 (PBS), 1.0 (VNP20009), 2.1 (VNPΔ-CA), 1.6 (VNPΔ-GA), and 2.4 (VNPΔ-CGA), showing an increasing trend in the VNPΔ-CGA group compared with the PBS and VNP20009 groups (Figure S10). The data suggested a trend toward macrophage remodeling after VNPΔ-CGA treatment. Given the increased APC abundance, we next examined cytotoxic CD8^+^ T cell infiltration through flow cytometry and immunofluorescence staining. Flow cytometric analysis showed that VNPΔ-CGA treatment increased the proportion of CD3^+^ CD8^+^ T cells in tumor tissues compared with PBS and the other bacterial treatment groups (Figures 5G and 5H). Consistently, CD8 immunofluorescence staining showed more CD8^+^ cells in the VNPΔ-CGA group, and quantitative analysis confirmed a significant increase in CD8^+^ T cells per field (Figures S11A and S11B). Together with the increased DC maturation observed after VNPΔ-CGA treatment, these results suggest that the dual-payload engineered bacteria promoted antigen presentation-related immune activation and enhanced cytotoxic T cell infiltration within the TME. Tumor cytokine levels were further measured to evaluate local inflammatory responses after bacterial treatment. Although some bacterial treatment groups showed a tendency toward increased TNF-α, IL-6, and IFN-γ levels in tumor tissues, no statistically significant differences were observed among the treatment groups (Figures S12A–S12C). However, IL-10 levels were increased after bacterial treatment (Figure S12D). Recent studies have shown that IL-10 may play a beneficial role in bacterial tumor immunotherapy by promoting bacterial persistence in tumors and participating in CD8^+^ T cell-related immune remodeling. Therefore, the elevated IL-10 level observed after VNPΔ-CGA treatment may reflect an IL-10-associated immune regulatory process induced by bacterial colonization within the TME.

**Figure 5:**
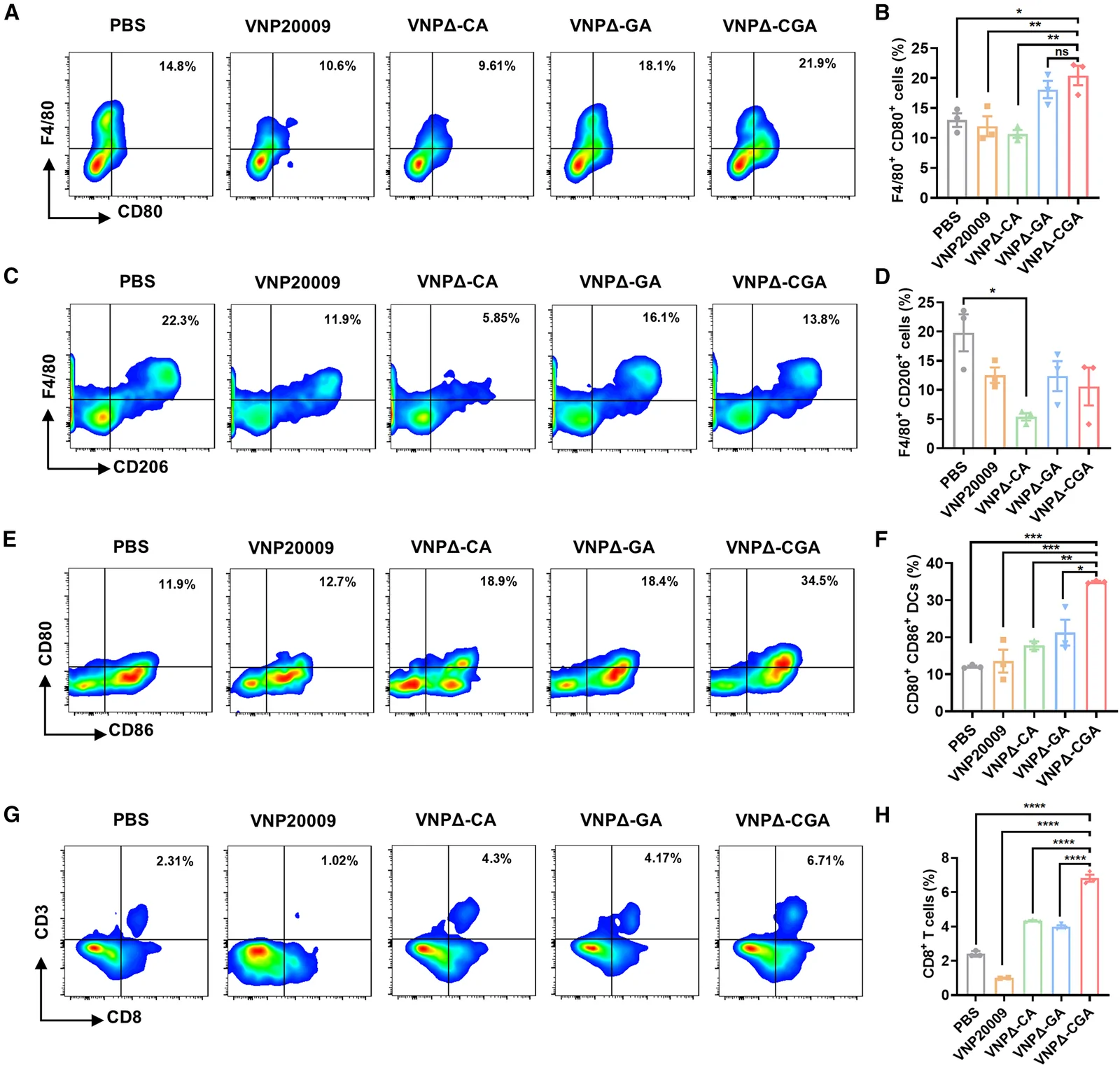
The immune cells infiltration in TME of different therapy groups (A and B) Flow cytometry analysis and quantification of the percentage of M1-like macrophages in tumors (gate out of CD45^+^ cells, mean ± SEM, *n* = 3). *p* values were analyzed by one-way ANOVA, ns, not significant; *p* > 0.05, ∗*p* ≤ 0.05, ∗∗*p* ≤ 0.01. (C and D) Flow cytometry analysis and quantification of the percentage of M2-like macrophages in tumors (gate out of CD45^+^ cells, mean ± SEM, *n* = 3). *p* values were analyzed by one-way ANOVA, ∗*p* ≤ 0.05. (E and F) Flow cytometry analysis and quantification of the percentage of mature DCs in tumors (gate out of CD11c^+^ cells, mean ± SEM, *n* = 3). *p* values were analyzed by one-way ANOVA, ∗*p* ≤ 0.05, ∗∗*p* ≤ 0.01, ∗∗∗*p* ≤ 0.001. (G and H) Flow cytometry analysis and quantification of the percentage of CD8^+^ T cells in tumors (gate out of CD45^+^ cells, mean ± SEM, *n* = 3). *p* values were analyzed by one-way ANOVA, ∗∗∗∗*p* ≤ 0.0001.

In conclusion, these findings demonstrate that VNPΔ-CGA enables localized delivery of CD47nb and GM-CSF. CD47nb is used to target and bind to the overexpressed CD47 on tumor cells, blocking the “do not eat me” signal of tumor cells and enhancing macrophage phagocytosis. Concurrently, GM-CSF is associated with increased M1-like macrophage characteristics within the TME and promotes DC maturation, thereby facilitating antigen presentation and promoting CD8^+^ T cell-mediated tumor eradication.

## Discussion

In this study, we developed an engineered *S. typhimurium* strain, VNPΔ-CGA, which integrates oxygen-dependent GM-CSF expression with bacterial density-dependent CD47nb expression for coordinated immune modulation within the TME. In tumor tissues, GM-CSF levels reached approximately 30.8 ng/g tissue, and VNPΔ-CGA accumulated at levels approximately 1,000–10,000-fold higher than those in healthy tissues. Based on this preferential tumor accumulation, the bacterial QS system was used to promote CD47nb expression preferentially in tumor-colonizing bacteria, which may help reduce unnecessary CD47 blockade on normal cells, such as erythrocytes.^41^ Together, these results support the potential of VNPΔ-CGA as an engineered bacterial strategy for tumor immunotherapy.

The antitumor activity of VNPΔ-CGA was mainly evaluated in the s.c. CT26 tumor model, which is a widely used syngeneic colorectal cancer model for immunotherapy studies and provides a practical platform for evaluating bacterial tumor colonization, therapeutic efficacy, and immune remodeling. In this model, VNPΔ-CGA showed a tendency to increase the ratio of M1-like to M2-like macrophages in the TME, promoted DC maturation, and increased CD8^+^ T cell infiltration. However, orthotopic tumors or tumors with distinct immune backgrounds may exhibit different anatomical and immunological features, and evaluation in additional tumor models would further help define the broader applicability of this strategy.

The survival analysis showed a moderate separation between VNPΔ-CGA and the single payload strains, with 25% of mice in the VNPΔ-CGA group remaining alive on day 36. This result suggests that although the current dual-payload design improved the therapeutic outcome to some extent, further refinement of the engineered bacterial platform may enhance its antitumor efficacy. Notably, VNPΔ-CGA treatment increased the infiltration of immune-activating cell populations within the TME, including M1-like macrophages, mature DCs (almost 20% higher than VNP20009) and CD8^+^ T cells, indicating that this engineered bacterial strategy can promote antitumor immune remodeling in the TME. Future optimization of this platform may focus on improving payload combinations or integrating complementary therapeutic modules to further enhance tumor inhibition. One possible direction is to increase the availability of tumor-associated antigens (TAAs) or tumor-specific antigens (TSAs) from tumor cells or harness engineered bacteria to produce TSAs,^42^ thus further boosting antitumor effect. Besides, GM-CSF has context-dependent roles in tumor immunity: relatively low-dose GM-CSF can enhance vaccine-induced immune responses, whereas higher doses may fail to improve immunity or even induce immunosuppressive effects, partly through the recruitment or expansion of myeloid suppressor cells.^31^ Therefore, local and regulated delivery of GM-CSF by engineered bacteria may be important for improving antitumor immune activation while reducing unwanted systemic or context-dependent effects.

### Limitations of the study

Future studies should address several aspects to further improve and evaluate this strategy. First, the antitumor efficacy of VNPΔ-CGA was evaluated mainly in s.c. syngeneic tumor models, and additional orthotopic or clinically relevant models are needed to assess its broader applicability. In addition, increasing the administrable dose of VNPΔ-CGA without exacerbating host toxicity remains an important goal. One possible strategy is to further improve tumor-selective bacterial survival by coupling bacterial viability to tumor-associated signals, such as hypoxia or elevated lactate levels. Moreover, whether limited availability of TSAs and TAAs constrains T cell activation remains to be determined. Increasing i.t. antigen availability may therefore represent a further direction for promoting activated T cell infiltration and improving antitumor efficacy.

## Resource availability

### Lead contact

For additional information and requests for resources and reagents, please contact Ying Zhou (zhouying@ecust.edu.cn).

### Materials availability

All materials generated in this study are availability through the lead contact.

### Data and code availability

• Data reported in this paper will be shared by the lead contact upon request.
• This paper does not report original code.
• All other items required to reanalyze the data reported in this paper is available from the lead contact upon request.

## Acknowledgments

This work was supported by the 10.13039/501100012166National Key Research and Development Program of China (2024YFA0917100) and the 10.13039/501100001809National Natural Science Foundation of China (32501359 and 22134003).

## Author contributions

B.-C.Y., Y.Z., and Z.-P.Z. conceptualized and supervised the project. X.-G.W. performed the main experiments. Y.-L.H., and X.-R.S. participated in the experiments. Z.-P.Z. and X.-G.W. wrote the manuscript. B.-C.Y., Y.Z., and Z.-P.Z. edited the manuscript.

## Declaration of interests

The authors declare no competing interests.

## STAR★Methods

### Key resources table

**Table:** 

REAGENT or RESOURCE	SOURCE	IDENTIFIER
**Antibodies**		

anti-Mouse IgG (H + L), HRP Conjugate	Transgen Biotech	Cat#HS201-01, RRID:AB_2629433
anti-DYKDDDDK Mouse Monoclonal Antibody	Transgen Biotech	Cat# HT201-01, RRID:AB_2927465
anti-HA Mouse Monoclonal Antibody	Transgen Biotech	Cat#HT301-01, RRID:AB_3076171
anti-mouse CD16/32	BioLegend	Cat# 156603, RRID:AB_2783137
anti-CD47-FITC	eBioscience	Cat#11-0471-82, RRID:AB_837105
anti-CD86-PE	Proteintech	Cat#PE-65068, RRID:AB_2883865
anti-CD80-FITC	Proteintech	Cat#FITC-65076, RRID:AB_2883762
anti-CD45^−^ CoraLite®594	Proteintech	Cat#CL594-65087, RRID:AB_3084658
anti-F4/80- eFluor™ 450	BioLegend	Cat#48-4801-82, RRID:AB_1548747
anti-CD11b- APC-Cyanine7	Proteintech	Cat#AY7-65055, RRID:AB_3672359
anti-CD206-APC	BioLegend	Cat#17-2061-82, RRID:AB_2637420
anti-CD11c-APC	Proteintech	Cat#APC-65130, RRID:AB_2883012
anti-CD3-FITC	Proteintech	Cat#CL488-65077, RRID:AB_2883163
anti-CD8-APC-Cyanine7	Proteintech	Cat#AY7-FcA98472-2, RRID:AB_3746715

**Experimental models: Organisms/strains**		

VNP20009	Lab storage	N/A
BL21	Transgen Biotech	Cat#CD601-02

**Chemicals, peptides, and recombinant proteins**		

DAP (2,6-diaminopimelic acid)	Sigma	Cat#33240
RIPA lysis buffer	Yeasen	Cat#20101ES60
Collagenase IV	Biotopped	Cat#CG6160C
DNase I	Biotopped	Cat#D6071C
Percoll	Yeasen	Cat#40501ES60

**Critical commercial assays**		

QuantiCyto® Mouse GM-CSF ELISA kit	NeoBioscience	Cat# EMC111.96
QuantiCyto® Mouse TNF-α ELISA kit	NeoBioscience	Cat# EMC102a.96
QuantiCyto® Mouse IL-6 ELISA kit	NeoBioscience	Cat# EMC004.96
QuantiCyto® Mouse IFN-γ ELISA kit	NeoBioscience	Cat# EMC101g.96
QuantiCyto® Mouse IL-10 ELISA kit	NeoBioscience	Cat# EMC005.48
Zombie R718™ Fixable Viability Kit	BioLegend	Cat#423115

**Experimental models: Cell lines**		

CT26	Cell Bank, Chinese Academy of Sciences	Cat# TCM37
RAW264.7	Lab storage	N/A

**Experimental models: Organisms/strains**		

BALB/c	Shanghai Model Organisms Center	Cat# SM-003

**Software and algorithms**		

GraphPad Prism 8	GraphPad	https://www.graphpad.com/
FlowJo 10.8.1	FlowJo, LLC	https://www.flowjo.com/
SnapGene 3.2.1	SnapGene	https://www.snapgene.com/

### Experimental model and study participant details

#### Ethics

Female BALB/c (six-week-old) were obtained from Shanghai Model Organisms Center, Inc. All animal procedures were performed in accordance with the Guidelines for Care and Use of Laboratory Animals of East China University of Science and Technology and the experiments were approved by the Animal Ethics Committee of East China University of Science and Technology (ECUST-21038).

#### Bacteria and cells culture

All bacterial strains were stored as glycerol stocks containing 50% glycerol at −80 °C. For bacterial culture, 10 μL of each glycerol stock was spread on the LB solid plate with relevant antibiotics: streptomycin (100 μg/mL), kanamycin (50 μg/mL), chloramphenicol (25 μg/mL) and/or ampicillin (100 μg/mL) culturing at 37 °C or 30 °C for 12 h. Colonies were picked out and inoculated to 700 μL LB liquid medium with corresponding antibiotics shaking in a microplate shaker (37 °C or 30 °C, 1000 rpm) for overnight. Afterward, the activated cultures were diluted 200-fold into fresh medium and further cultured to the logarithmic phase.

Cells were maintained at 37 °C in a 5% CO_2_ humidified atmosphere. CT26 cells (mouse colon carcinoma, CRL-2638) purchased from Cell Bank, Chinese Academy of Sciences were cultured in 1640 medium supplemented with 10% fetal bovine serum (FBS) and 100 U/mL penicillin-streptomycin. RAW264.7 cells were cultured in DMEM medium with 10% fetal bovine serum (FBS) and 100 U/mL penicillin-streptomycin.

#### Establishment of CT26-bearing tumor mouse model

Female BALB/c mice, 6 weeks old, were purchased from Shanghai Model Organisms Center, Inc. (Cat# SM-003) and acclimatized for one week before experiments. To establish the CT26 subcutaneous tumor model, 5 × 10^5^ CT26 cells in 100 μL PBS were subcutaneously injected into each mouse for therapeutic efficacy experiments, whereas 1 × 10^6^ CT26 cells in 100 μL PBS were used for the survival curve experiment. When tumor volumes reached 50–100 mm^3^, mice were randomly assigned to different treatment groups. Day 0 was defined as the day when mice first received PBS or bacterial treatment. Engineered bacteria in the logarithmic growth phase were washed twice with cold PBS and resuspended in PBS. Mice received i.v. injections of PBS or bacteria through the tail vein at a dose of 2 × 10^6^ CFU in 100 μL PBS per mouse on day 0 and day 6. Tumor volume and body weight were measured every two days. Tumor volume was calculated using the following formula: volume = (length × width^2^)/2.^43^ For tumor growth and tumor weight measurements, each group contained five mice. For survival analysis, each group contained eight mice. For immune cell profiling by flow cytometry, each group contained three mice. For serum cytokine and liver function analyses, each group contained three mice. For bacterial colonization analysis in tumors and major organs, each group contained five mice.

### Method details

#### Construction of strains and plasmids

All strains constructed in this study are listed in Table S1 (supplemental information), crucial primers and relevant gene sequences are listed in Table S2 λ-Red recombination method was used to knock out the *asd* gene from VNP20009 by *CmR*.^44^ Correct gene-deletion clones were selected by LB agar plates with 25 μg/mL chloramphenicol. Plasmids were constructed by an origin replication of pSC101. Indeed, on account of the resistance to *SmR* of VNP20009, the *SmR* resistance gene of pSC101 was replaced by *KanR*. The MISSSSIS-GM-CSF-HA fragment was synthesized by Tsingke. Genes sequences encoding OmpA, OmpC, OmpF, InfB peptides, *asd* and oxygen dependent promoters and backbones used in this study are amplified by polymerase chain reaction (PCR) and linked together by overlap PCR or homologous recombination. Both luxR-OmpA-Flag-CD47nb-luxI and yfiD-*m*-OmpF-infB-GM-CSF-HA fragments were obtained by PCR and overlap PCR. The fragments were linked with pSC101 backbone by homologous recombination. The resulting plasmids were named pWT-C, pWT-G, pWT-CG, separately. With *asd* gene insertion, they were named pWT-CA, pWT-GA, pWT-CGA, while letters C, G and A were on behalf of CD47nb, GM-CSF, *asd* genes, respectively. All plasmids were transformed into VNP20009 or VNP20009Δ*asd* by electroporation.

#### Expression and purification of CD47nb and GM-CSF

The plasmid of pET-28a-CD47nb or pET-28a-GM-CSF were transferred into *E. coli* BL21 strain respectively. The proteins were expressed and purified according to standard protocols in *E. coli* BL21 strain. Briefly, the bacteria were first inoculated into the 5 mL LB medium and cultured overnight at 37 °C shaker. The next day, the bacteria solution was inoculated into fresh LB medium and cultured to the logarithmic growth phase. After that, 1 mM IPTG was added into the culture to induce target protein expression for 16 h at 18 °C. Then the bacteria were collected and washed with PBS by centrifugation (8000 rpm, 10 min) before crushed by the ultrasonic cell disruptor. The supernatant containing the whole protein was collected by centrifugation (10000 rpm, 10 min). The whole protein was loaded onto the nickel column equilibrated by 10 mM imidazole. The column was washed with 20 mM imidazole to remove impurities, and the target protein was eluted with 250 mM imidazole and collected into a centrifuge tube. The ultrafiltration tube was washed with PBS (4500 rpm, 10 min), and the whole protein solution was added into the ultrafiltration tube to concentrate. Finally, the concentrated target protein was obtained.

#### Measurement of luminescence and OD_600_

To measure the luminescence signals of bacteria under aerobic and anaerobic environment, the activated bacteria were inoculated into two 96-well plates with 200 μL fresh LB media respectively culturing to the logarithmic phase. Subsequently, one of them was transferred into anaerobic environment inducing for 8 h. The another was still shaking in a microplate shaker (37 °C, 1000 rpm) to stay aerobic. After that, the bacteria were transferred into 96-well white plate to test the OD_600_ and luminescence signals by the microplate reader (BioTek Instruments, Winooski, VT, USA). RLU/OD_600_ represented the normalized luminescence signals.

#### Western blotting

For detecting CD47nb, the activated bacteria were inoculated into fresh media (1:200 dilution) culturing for about 12 h. For detecting GM-CSF, the activated bacteria were inoculated into fresh media (1:200 dilution) culturing to the logarithmic phase, then induced under anaerobic environment for about 8 h. Afterward, pellets and supernatants were obtained by centrifugation. Pellets were resuspended with PBS and supernatants were concentrated by ultrafiltration. The induced supernatant was stored at −80 °C. Pellets solution and supernatants were mixed with 6 × protein loading buffer (TransGen Biotech) and incubated for 10 min at 95 °C. Samples were loaded into different well of a 12.5% SDS-PAGE gel (Beyotime biotechnology), followed by transferring to PVDF membranes (Merck) and WB test was the performed in line with the standard protocol, in which anti-HA mouse and anti-Flag mouse antibody (1:5000 dilution) (TransGen Biotech) served as primary antibodies were used to detect GM-CSF and CD47nb, and the horseradish peroxidase (HRP) conjugated goat anti-mouse IgG (1:5000 dilution) (TransGen Biotech) was utilized as the second antibody.

#### Enzyme linked immunosorbent assay (ELISA)

For CD47nb detection: purified CD47nb with a series of dilution gradient and the obtained supernatant above were incubated in a 96-well ELISA plate for 1 h at 37 °C before blocking with 5% bovine serum albumin (BSA) for 1 h at 37 °C. Then anti-Flag mouse antibody (1:2000 dilution) was added into the 96-well ELISA plate staying for 1 h at 37 °C. After that, the horseradish peroxidase (HRP) conjugated goat anti-mouse IgG was added incubating for 1 h at 37 °C. Finally, the TMB was utilized for coloration and 2 M H_2_SO_4_ for termination before OD_450_ was tested by the microplate reader. It is necessary to wash the plate three times with PBST between each step. For GM-CSF detection: all supernatant samples were detected with QuantiCyto Mouse GM-CSF ELISA kit (NeoBioscience) followed with the instruction of manufacturer.

#### Assessment of the bioactivity of CD47nb and GM-CSF secreted by engineered bacteria

All supernatant samples were filtered through the 0.22 μm filter membrane. For CD47nb: CT26 cells were seeded into a 24-well plate for 12 h. Then, supernatants were added and incubated with CT26 cells overnight at 37 °C. After that, cells were harvested and washed twice (500×*g*, 5 min) with the flow cytometry buffer (0.5% BSA in PBS). Subsequently, the cells were incubated with FITC anti-CD47 antibody (eBioscience, 11-0471-82) for 30 min at 4 °C. After the cells were washed twice again, they were analyzed by flow cytometry. For GM-CSF: RAW264.7 cells were seeded into a 24-well plate for 12 h. Then, diluted supernatants were added and incubated with RAW264.7 cells for 48 h at 37 °C. After that, cells were harvested and washed twice (500×*g*, 5 min) with the flow cytometry buffer. Subsequently, the cells were incubated with PE anti-CD86 antibody (Proteintech, PE-65068) and FITC anti-CD80 antibody (Proteintech, FITC-65076) for 30 min at 4 °C. After the cells were washed twice again, they were analyzed by CytoFLEX S (Beckman Coulter).

#### Quantification of bacteria in tumor and healthy tissues of tumor-bearing mice

Mice were injected intravenously with 100 μL of engineered bacteria and sacrificed at specific time points (24 h or end of treatment). The former one was to examine the engineered plasmid stability of bacteria in mice. Another one was to characterize the bacteria distribution in tumor and healthy tissues. Tumors and healthy tissues were immerged in 500 μL of PBS and homogenized with the tissue homogenizer (Servicebio). The homogenized samples were diluted and spread on the LB agar plate with corresponding antibiotics, after which colonies were counted after 12 h. Bacterial titers (CFU/g tissue) were calculated based on colony counts, dilution ratio and the tissue weight.

#### WB to detect CD47nb in tumors secreted by engineered bacteria

Tumor tissues were homogenized in RIPA lysis buffer (Yeasen) and then centrifuged to obtain the supernatants. The supernatants were mixed with 6 × protein loading buffer (TransGen Biotech) and incubated for 10 min at 95 °C. The following steps were same as those described above.

#### ELISA to detect GM-CSF in tumors secreted by engineered bacteria

Tumor tissues were homogenized in PBS and then centrifuged to obtain the supernatants. The supernatants were detected with QuantiCyto Mouse GM-CSF ELISA kit (NeoBioscience).

#### Immunohistochemistry analysis of tumor tissues

Sample sections were fixed on slides (5 μm) and stained with hematoxylin and eosin. The immunohistochemistry was accomplished by Servicebio. Photos were taken by an inverted microscope.

#### Intra-tumoral cytokine analysis

Tumor tissues were homogenized in PBS and then centrifuged to obtain the supernatants. The supernatants were detected with QuantiCyto Mouse TNF-α ELISA kit, QuantiCyto Mouse IL-6 ELISA kit, QuantiCyto Mouse IFN-γ ELISA kit and QuantiCyto Mouse IL-10 ELISA kit (NeoBioscience) followed with the instructions of manufacturer.

#### Analysis of immune cells infiltration in tumor tissues by flow cytometry

For immune cell profiling, CT26 tumor-bearing mice received PBS or bacteria intravenously on day 0 and day 6. On day 12 after the initial administration, mice were sacrificed and tumor tissues were collected and immersed in 2 mL of ice-cold RPMI 1640 medium and then cut into 1–3 mm pieces with a scalpel in the Petri dish. The tissues were subsequently digested with 1 mg/mL collagenase type IV (Absin) and 50 μg/mL DNase I (Biotopped) and incubated for 1 h at 37 °C. Then the single cell suspensions were obtained by filtering the digested tissue solutions with 70 μm cell strainers. Further, the cell debris was removed by the density gradient centrifugation based on different concentration of percoll solutions (Yeasen). Finally, the cells were resuspended with flow cytometry buffer. Then the live cells were distinguished with zombie R718 Fixable Viability Kit (BioLegend, 423115). Moreover, cells were incubated with Fc blocking buffer (BioLegend, 156603) for 15 min at 4 °C to avoid nonspecific binding. The samples were then stained for 30 min on ice with specific antibodies. CoraLite594 anti-mouse CD45 antibody (Proteintech, CL594-65087), eFluor 450 anti-F4/80 antibody (Biolegend, 48–4801-82), APC-Cyanine7 anti-mouse CD11b antibody (Proteintech, AY7-65055), PE anti-CD86 antibody (Proteintech, PE-65068) and FITC anti-CD80 antibody (Proteintech, FITC-65076) are to determine the M1 type macrophages; CoraLite594 anti-mouse CD45 antibody (Proteintech, CL594-65087), eFluor 450 anti-F4/80 antibody (Biolegend, 48-4801-82), APC-Cyanine7 anti-mouse CD11b antibody (Proteintech, AY7-65055), APC anti-mouse CD206 antibody (Biolegend, 17-2061-82) are to characterize M2 type macrophages; CoraLite594 anti-mouse CD45 antibody (Proteintech, CL594-65087), APC anti-mouse CD11c antibody (Proteintech, APC-65130), PE anti-CD86 antibody (Proteintech, PE-65068) and FITC anti-CD80 antibody (Proteintech, FITC-65076) are to characterize dendritic cells (DCs); CoraLite594 anti-mouse CD45 antibody (Proteintech, CL594-65087), FITC anti-mouse CD3 antibody (Proteintech, CL488-65077), APC-Cyanine7 anti-mouse CD8 (Proteintech, AY7-FcA98472-2) are to characterize CD8^+^ T cells. Among them, cells needed to be fixed with flow cytometry fix buffer (Proteintech, PF00016) and permeabilized with flow cytometry permeabilization buffer (Proteintech, PF00017) before incubation with APC anti-mouse CD206 antibody (Biolegend, 17-2061-82). After that, the samples were washed and resuspended with the flow cytometry buffer or perm buffer and analyzed through the CytoFLEX S (Beckman Coulter). Data were analyzed by FlowJo software (Becton, Dickinson & Company, USA) in which at least 10^5^ cellular events were recorded per sample.

### Quantification and statistical analysis

Statistical analyses and graphical output of the data were performed with the computer program Prism (GraphPad Prism8). The data were reported as the means ± SEMs; Student’s *t* test was used to analyze significant differences between two groups, one-way ANOVA with Tukey’s multiple comparisons post-test was employed for data analysis involving three groups or more. two-way ANOVA with Tukey’s multiple comparisons test was used for data involving two factors. Two-way repeated-measures ANOVA was used for longitudinal tumor growth analysis. Survival curves were analyzed using the Kaplan-Meier method and compared using the log rank test. ns *p* > 0.05, ∗*p* ≤ 0.05, ∗∗*p* ≤ 0.01, ∗∗∗*p* ≤ 0.001, and ∗∗∗∗*p* ≤ 0.0001.

## References

[1] Yang E.-L., Wang W.-Y., Liu Y.-Q., Yi H., Lei A., Sun Z.-J. (2025). Tumor-Targeted Catalytic Immunotherapy. Adv. Mater..

[2] Waldman A.D., Fritz J.M., Lenardo M.J. (2020). A guide to cancer immunotherapy: from T cell basic science to clinical practice. Nat. Rev. Immunol..

[3] Pittet M.J., Di Pilato M., Garris C., Mempel T.R. (2023). Dendritic cells as shepherds of T cell immunity in cancer. Immunity.

[4] Whiteside T.L. (2008). The tumor microenvironment and its role in promoting tumor growth. Oncogene.

[5] Yuan X., Xiao Y., Yu D. (2025). Turn cold tumors hot by reprogramming the tumor microenvironment. Nat. Biotechnol..

[6] Gurbatri C.R., Arpaia N., Danino T. (2022). Engineering bacteria as interactive cancer therapies. Science.

[7] Felfoul O., Mohammadi M., Taherkhani S., de Lanauze D., Zhong Xu Y., Loghin D., Essa S., Jancik S., Houle D., Lafleur M. (2016). Magneto-aerotactic bacteria deliver drug-containing nanoliposomes to tumour hypoxic regions. Nat. Nanotechnol..

[8] Kashfi K., Kannikal J., Nath N. (2021). Macrophage Reprogramming and Cancer Therapeutics: Role of iNOS-Derived NO. Cells.

[9] Tseng D., Volkmer J.-P., Willingham S.B., Contreras-Trujillo H., Fathman J.W., Fernhoff N.B., Seita J., Inlay M.A., Weiskopf K., Miyanishi M., Weissman I.L. (2013). Anti-CD47 antibody–mediated phagocytosis of cancer by macrophages primes an effective antitumor T-cell response. Proc. Natl. Acad. Sci. USA.

[10] Tang Z., Zhong M.-C., Qian J., Galindo C.C., Davidson D., Li J., Zhao Y., Hui E., Veillette A. (2023). CD47 masks pro-phagocytic ligands in cis on tumor cells to suppress antitumor immunity. Nat. Immunol..

[11] Kumagai S., Itahashi K., Nishikawa H. (2024). Regulatory T cell-mediated immunosuppression orchestrated by cancer: towards an immuno-genomic paradigm for precision medicine. Nat. Rev. Clin. Oncol..

[12] Li K., Shi H., Zhang B., Ou X., Ma Q., Chen Y., Shu P., Li D., Wang Y. (2021). Myeloid-derived suppressor cells as immunosuppressive regulators and therapeutic targets in cancer. Signal Transduct. Target. Ther..

[13] Xiang X., Wang J., Lu D., Xu X. (2021). Targeting tumor-associated macrophages to synergize tumor immunotherapy. Signal Transduct. Target. Ther..

[14] Niu L., Deng Z., Jin Y., Guan N., Ye H. (2026). Engineering oncolytic bacteria as precision cancer therapeutics: design principles, therapeutic strategies, and translational perspectives. Protein Cell.

[15] Xie X.-T., Guan M., Cheng K., Li Y., Zhang B., Zhou Y.-T., Tan L.-F., Dong P.-S., Chen S., Liu B. (2025). Programmable engineered bacteria as sustained-releasing antibody factory in situ for enhancing tumor immune checkpoint therapy. Sci. Adv..

[16] Sun M., Yang J., Li L., Li Y., Li W., Ding J. (2025). Engineered bacteria potentiate cancer immunotherapy. Chin. Chem. Lett..

[17] Nguyen D.-H., You S.-H., Ngo H.T.-T., Van Nguyen K., Tran K.V., Chu T.-H., Kim S.-y., Ha S.-J., Hong Y., Min J.-J. (2024). Reprogramming the tumor immune microenvironment using engineered dual-drug loaded Salmonella. Nat. Commun..

[18] Tan W., Duong M.T.-Q., Zuo C., Qin Y., Zhang Y., Guo Y., Hong Y., Zheng J.H., Min J.-J. (2022). Targeting of pancreatic cancer cells and stromal cells using engineered oncolytic Salmonella typhimurium. Mol. Ther..

[19] Zhou T., Wu J., Tang H., Liu D., Jeon B.-H., Jin W., Wang Y., Zheng Y., Khan A., Han H., Li X. (2024). Enhancing tumor-specific recognition of programmable synthetic bacterial consortium for precision therapy of colorectal cancer. NPJ Biofilms Microbiomes.

[20] Zou Z.-P., Wang X.-G., Shi X.-R., Sun S.-T., Mi J., Zhang X.-P., Yin B.-C., Zhou Y., Ye B.-C. (2025). Self-Adjusting Engineered Probiotic for Targeted Tumor Colonization and Local Therapeutics Delivery. Adv. Sci..

[21] Savage T.M., Vincent R.L., Rae S.S., Huang L.H., Ahn A., Pu K., Li F., de los Santos-Alexis K., Coker C., Danino T., Arpaia N. (2023). Chemokines expressed by engineered bacteria recruit and orchestrate antitumor immunity. Sci. Adv..

[22] Yang Y., Wang Y., Zeng F., Chen Y., Chen Z., Yan F. (2024). Ultrasound-visible engineered bacteria for tumor chemo-immunotherapy. Cell Rep. Med..

[23] Chowdhury S., Castro S., Coker C., Hinchliffe T.E., Arpaia N., Danino T. (2019). Programmable bacteria induce durable tumor regression and systemic antitumor immunity. Nat. Med..

[24] Gurbatri C.R., Lia I., Vincent R., Coker C., Castro S., Treuting P.M., Hinchliffe T.E., Arpaia N., Danino T. (2020). Engineered probiotics for local tumor delivery of checkpoint blockade nanobodies. Sci. Transl. Med..

[25] Lv M., Guo S., Zhang X., Zou Y., Chen Q., Zang C., Huang S., Hu Y., Wang Y., Wang Q., Zhong J. (2023). Attenuated Salmonella-delivered PD-1 siRNA enhances the antitumor effects of EZH2 inhibitors in colorectal cancer. Int. Immunopharmacol..

[26] Camacho E.M., Mesa-Pereira B., Medina C., Flores A., Santero E. (2016). Engineering Salmonella as intracellular factory for effective killing of tumour cells. Sci. Rep..

[27] Flentie K., Kocher B., Gammon S.T., Novack D.V., McKinney J.S., Piwnica-Worms D. (2012). A bioluminescent transposon reporter-trap identifies tumor-specific microenvironment-induced promoters in Salmonella for conditional bacterial-based tumor therapy. Cancer Discov..

[28] Swofford C.A., Van Dessel N., Forbes N.S. (2015). Quorum-sensing Salmonella selectively trigger protein expression within tumors. Proc. Natl. Acad. Sci. USA.

[29] Zou Z.-P., Du Y., Fang T.-T., Zhou Y., Ye B.-C. (2023). Biomarker-responsive engineered probiotic diagnoses, records, and ameliorates inflammatory bowel disease in mice. Cell Host Microbe.

[30] Luo X., Li Z., Lin S., Le T., Ittensohn M., Bermudes D., Runyab J.D., Shen S.Y., Chen J., King I.C., Zheng L.M. (2001). Antitumor effect of VNP20009, an attenuated Salmonella, in murine tumor models. Oncol. Res..

[31] Jeon H.J., Lim D., So E., Kim S., Jeong J.-H., Song M., Lee H.-J. (2024). Controlling tumor progression and recurrence in mice through combined treatment with a PD-L1 inhibitor and a designer Salmonella strain that delivers GM-CSF. Acta Pharm. Sin. B.

[32] Wessendarp M., Watanabe-Chailland M., Liu S., Stankiewicz T., Ma Y., Kasam R.K., Shima K., Chalk C., Carey B., Rosendale L.-R. (2022). Role of GM-CSF in regulating metabolism and mitochondrial functions critical to macrophage proliferation. Mitochondrion.

[33] Hong I.-S. (2016). Stimulatory versus suppressive effects of GM-CSF on tumor progression in multiple cancer types. Exp. Mol. Med..

[34] van de Laar L., Coffer P.J., Woltman A.M. (2012). Regulation of dendritic cell development by GM-CSF: molecular control and implications for immune homeostasis and therapy. Blood.

[35] Spiro S., Guest J.R. (1990). FNR and its role in oxygen-regulated gene expression in Escherichia coli. FEMS Microbiol. Rev..

[36] Wichmann J., Behrendt G., Boecker S., Klamt S. (2023). Characterizing and utilizing oxygen-dependent promoters for efficient dynamic metabolic engineering. Metab. Eng..

[37] Lin Z., Meng F., Ma Y., Zhang C., Zhang Z., Yang Z., Li Y., Hou L., Xu Y., Liang X., Zhang X. (2024). In situ immunomodulation of tumors with biosynthetic bacteria promote anti-tumor immunity. Bioact. Mater..

[38] Tumas S., Meldgaard T.S., Vaaben T.H., Suarez Hernandez S., Rasmussen A.T., Vazquez-Uribe R., Hadrup S.R., Sommer M.O.A. (2023). Engineered E. coli Nissle 1917 for delivery of bioactive IL-2 for cancer immunotherapy. Sci. Rep..

[39] Hansted J.G., Pietikäinen L., Hög F., Sperling-Petersen H.U., Mortensen K.K. (2011). Expressivity tag: A novel tool for increased expression in Escherichia coli. J. Biotechnol..

[40] Jin Y., Fu L. (2023). Engineer a double team of short-lived and glucose-sensing bacteria for cancer eradication. Cell Rep. Med..

[41] Maute R., Xu J., Weissman I.L. (2022). CD47-SIRPα-targeted therapeutics: status and prospects. Immunooncol. Technol..

[42] Redenti A., Im J., Redenti B., Li F., Rouanne M., Sheng Z., Sun W., Gurbatri C.R., Huang S., Komaranchath M. (2024). Probiotic neoantigen delivery vectors for precision cancer immunotherapy. Nature.

[43] Zhao M., Yang M., Li X.-M., Jiang P., Baranov E., Li S., Xu M., Penman S., Hoffman R.M. (2005). Tumor-targeting bacterial therapy with amino acid auxotrophs of GFP-expressing Salmonella typhimurium. Proc. Natl. Acad. Sci. USA.

[44] Zou Z.-P., Ye B.-C. (2020). Long-Term Rewritable Report and Recording of Environmental Stimuli in Engineered Bacterial Populations. ACS Synth. Biol..

