# Supplementary material for: In situ-activated engineered bacteria release combined dual-therapeutic proteins for tumor immunotherapy
Source: iScience. 2026 Nov 2;29(11):118090. doi: 10.1016/j.isci.2026.118090 (PMC13755573; doi:10.1016/j.isci.2026.118090)
Supplement: Supplementary file 1 [file mmc1.pdf]

iScience, Volume *29*

Supplemental information

In situ-activated engineered bacteria release combined dual-therapeutic proteins for tumor immunotherapy

Xin-Ge Wang, Yue-Lan Huang, Xuan-Ren Shi, Zhen-Ping Zou, Ying Zhou, and Bang-Ce Ye

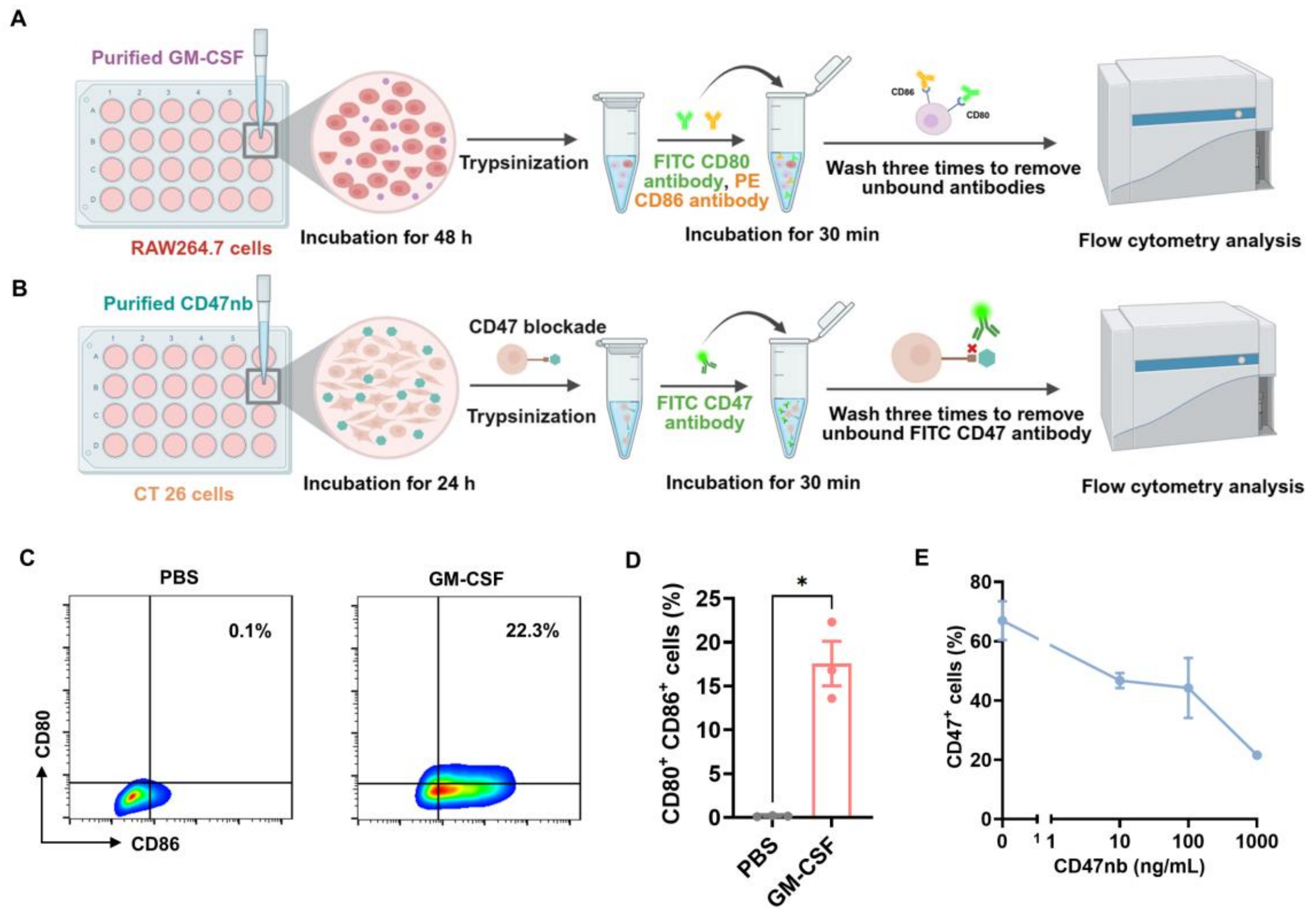

Figure S1. Functional validation of purified CD47nb and GM-CSF.

(A-B) Flowchart of the in vitro functional verification GM-CSF and CD47nb.

(C-D) Flow cytometry analysis and quantification of percentage of M1-like macrophages ($CD80^+$ $CD86^+$ cells) after RAW264.7 cells were incubated with PBS and purified GM-CSF protein for 48 h (mean ± SEM, *n*=3). *P* values were analyzed by independent t test, $*p \leq 0.05$.

(E) Change in the proportion of $CD47^+$ CT26 cells after incubating with serial concentrations of CD47nb (0 ng/mL, 10 ng/mL, 100 ng/mL, 1000 ng/mL) for 24 h (mean ± SEM, *n*=3).

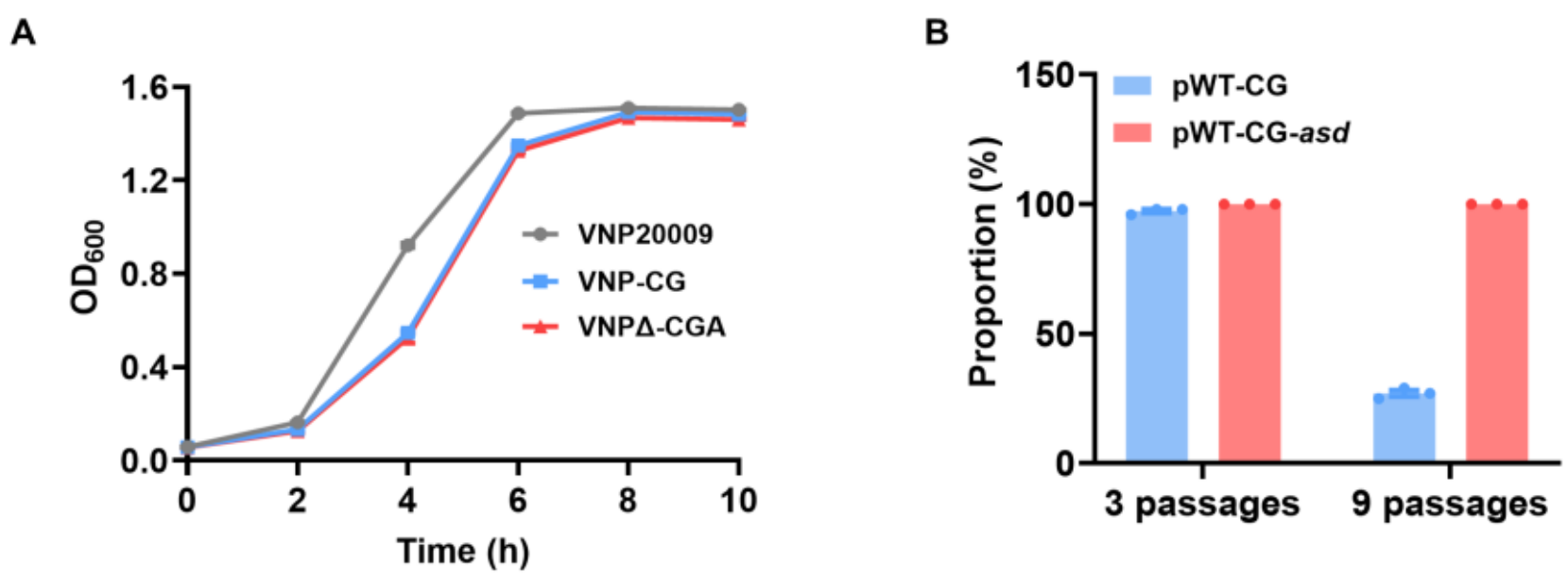

Figure S2. Verification of the plasmid balanced-lethal system.

(A) Growth curves of VNP20009, VNP-CG (without *asd* based plasmid balanced system) and VNPΔ-CGA (with *asd* based plasmid balanced system) within 10 h in the liquid LB media (mean ± SEM, *n*=3).

(B) Engineered plasmid retention rate in VNP-CG and VNPΔ-CGA when passaged to the third and ninth generations (mean ± SEM, *n*=3).

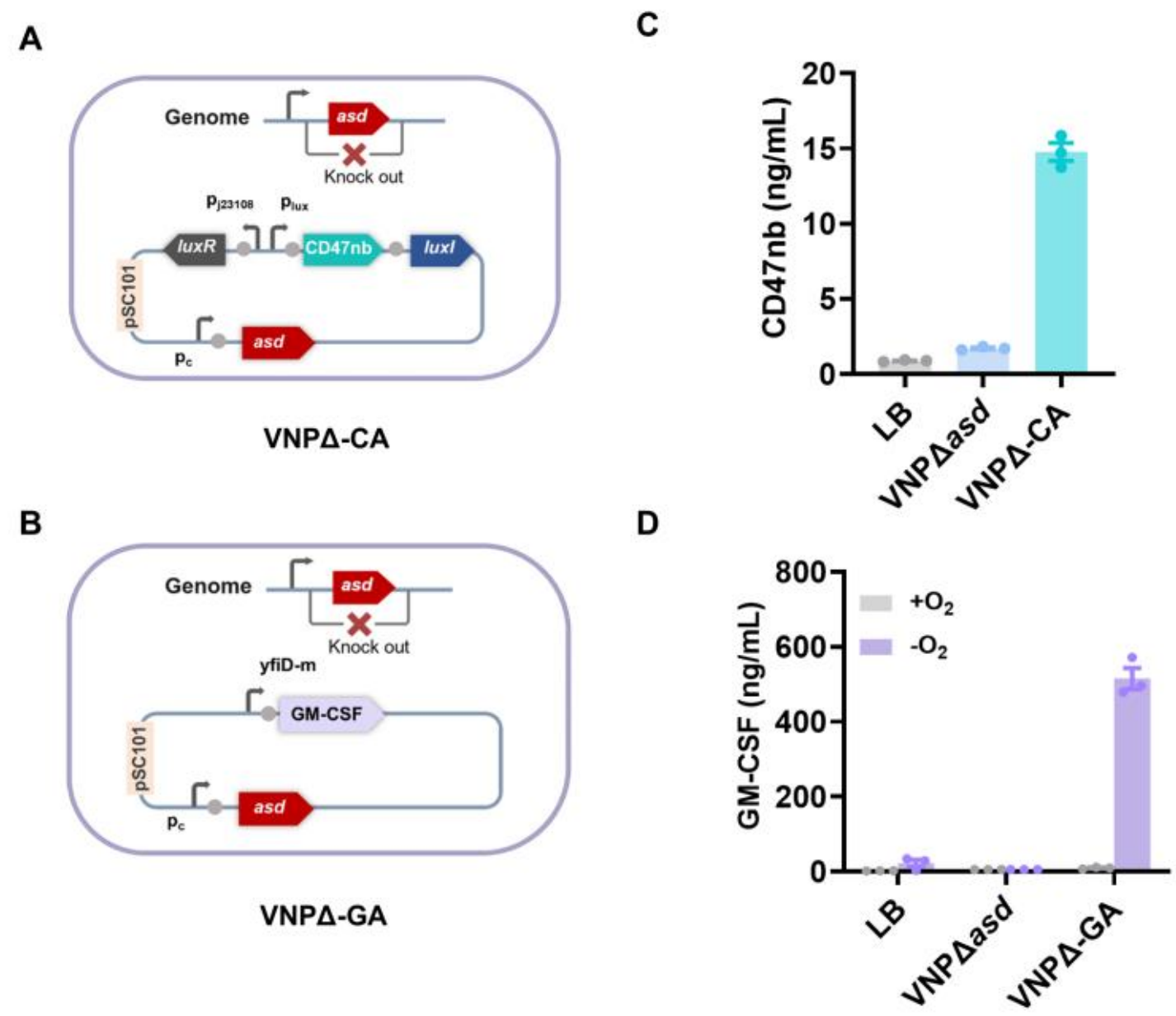

Figure S3. Construction and characterization of VNPΔ-CA and VNPΔ-GA.

(A) Schematic of the engineered plasmid containing the *lux* QS system-regulated CD47nb expression module and the *asd*-based balanced-lethal system module.

(B) Schematic of the engineered plasmid containing the hypoxia-responsive GM-CSF expression module and the *asd*-based balanced-lethal system module.

(C) CD47nb concentrations in culture supernatants of VNPΔ*asd* and VNPΔ-CA after 12 h culturing through ELISA with anti-Flag antibody (mean ± SEM, n=3).

(D) GM-CSF concentrations in culture supernatants of VNPΔ*asd* and VNPΔ-CA after 12 h culturing through ELISA with anti-HA antibody (mean ± SEM, n=3).

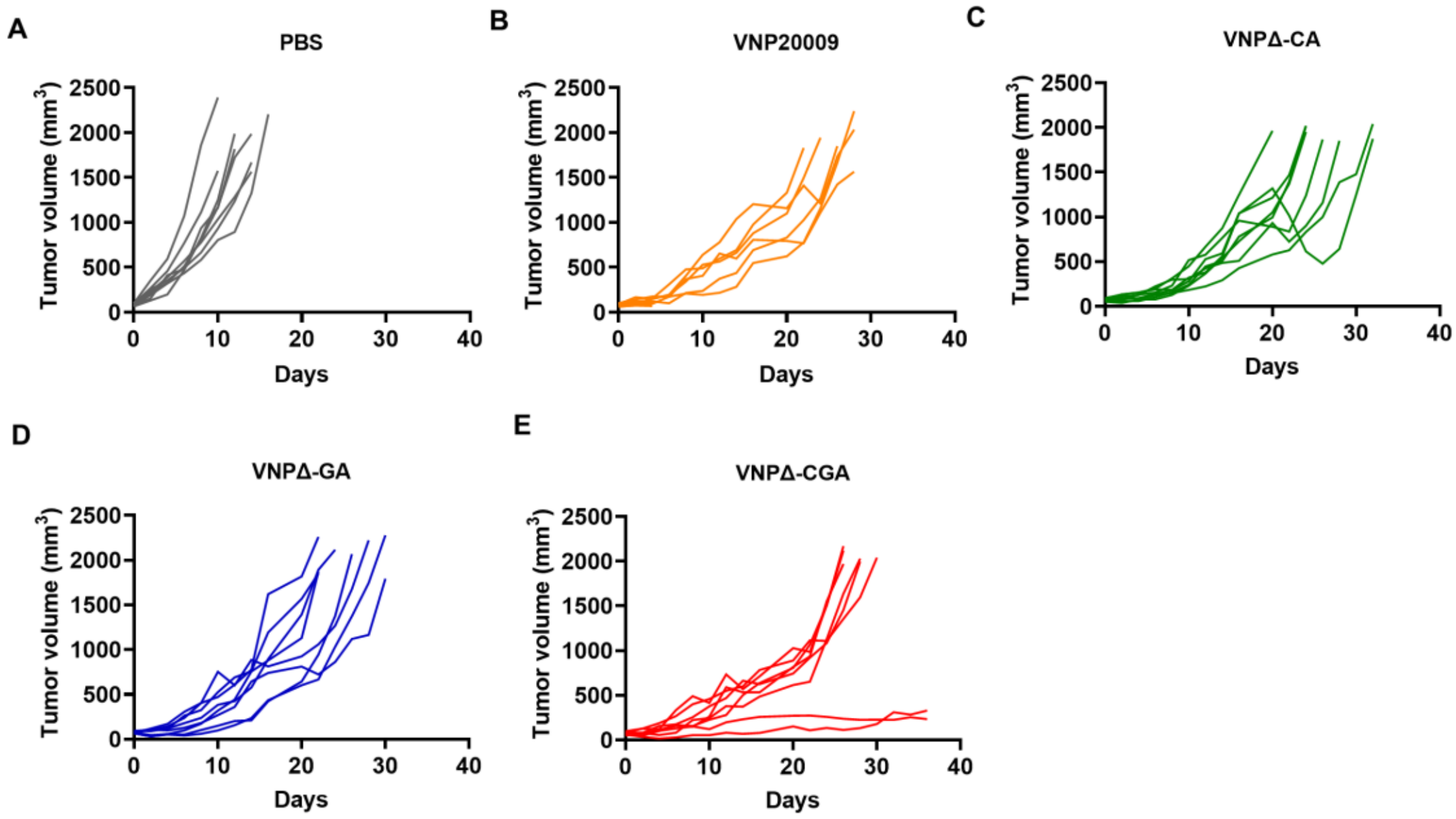

Figure S4. (A-E) Tumor growth curves of every single CT26 subcutaneous tumor-bearing mouse in different treatment groups (A: PBS; B: VNP20009; C: VNPΔ-CA; D: VNPΔ-GA; E: VNPΔ-CGA).

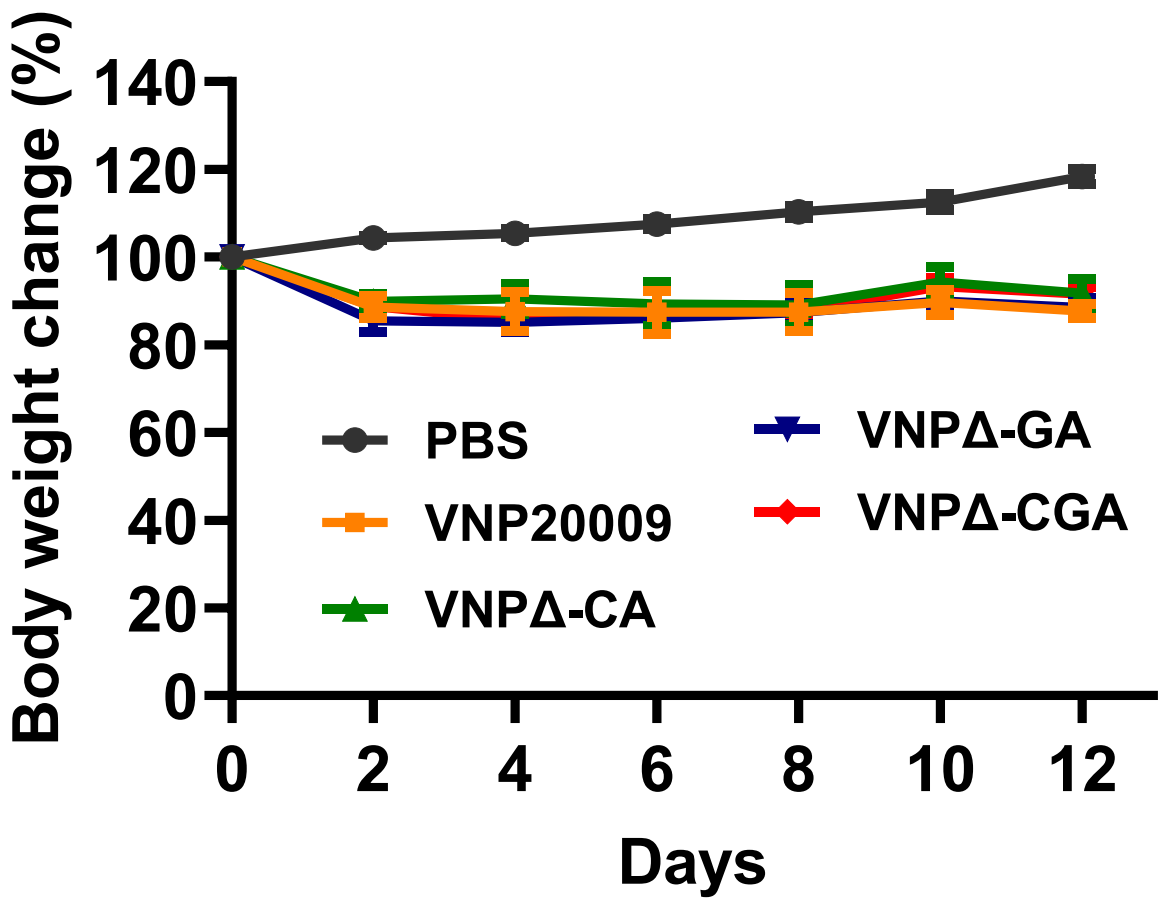

Figure S5. Average body weight changes of CT26 tumor-bearing mice in different treatment groups within 12 days after bacterial injection (mean ± SEM, *n*=5).

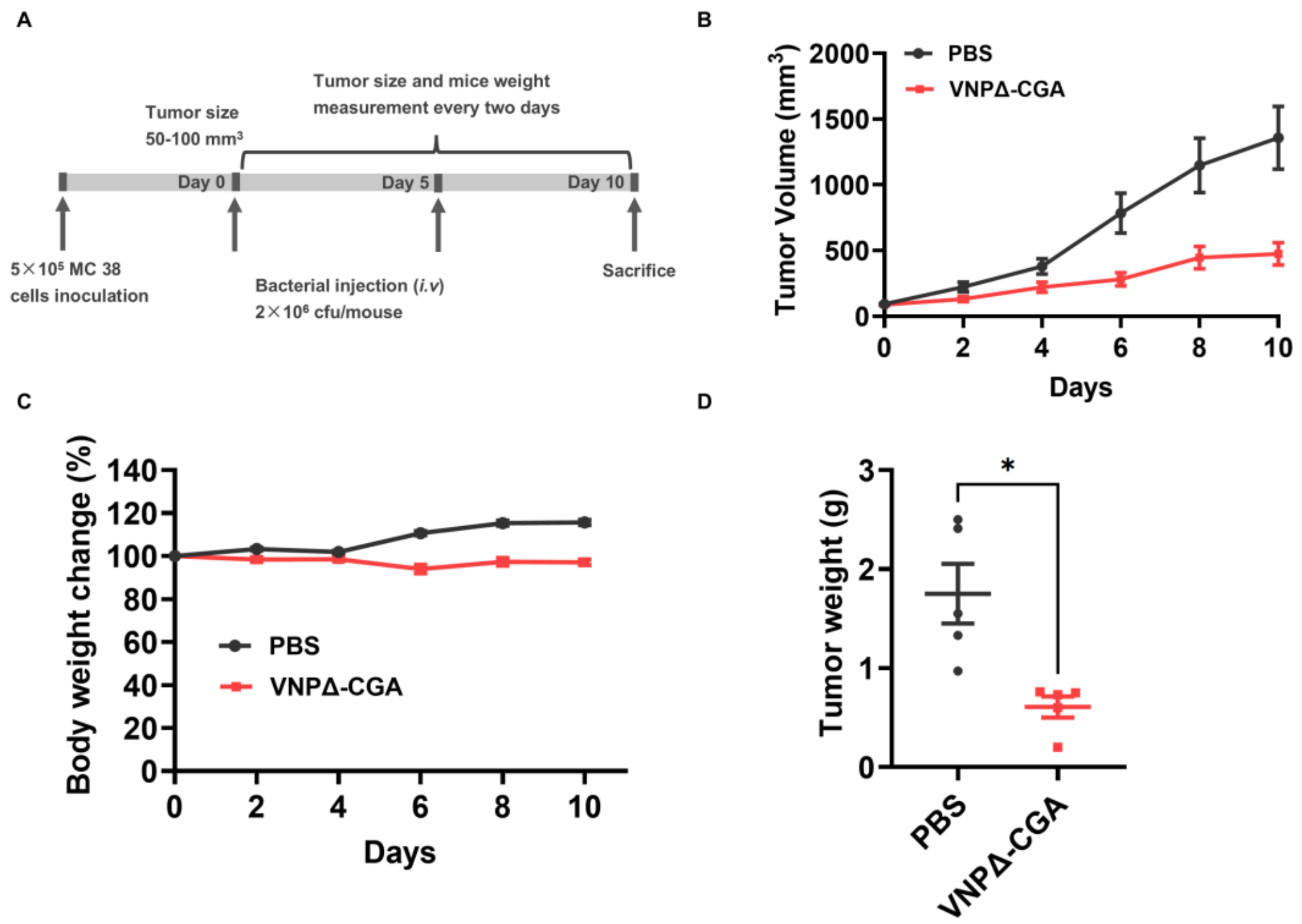

Figure S6. In vivo antitumor efficacy of VNPΔ-CGA treatment in MC38 tumor-bearing mice. (A) Schematic illustration of the experimental design for evaluating the therapeutic efficacy of VNPΔ-CGA.

(B) Tumor growth curves of MC38 tumor-bearing mice treated with PBS or VNPΔ-CGA. Statistical analysis was performed using two-way repeated-measures ANOVA followed by Sidak's multiple comparisons test (mean ± SEM, $n = 5$).

(C) Relative body weight changes of mice during the treatment period (mean ± SEM, $n = 5$).

(D) Tumor weights collected at the experimental endpoint (mean ± SEM, $n = 5$). *P* values were analyzed by independent t test, $*p \leq 0.05$.

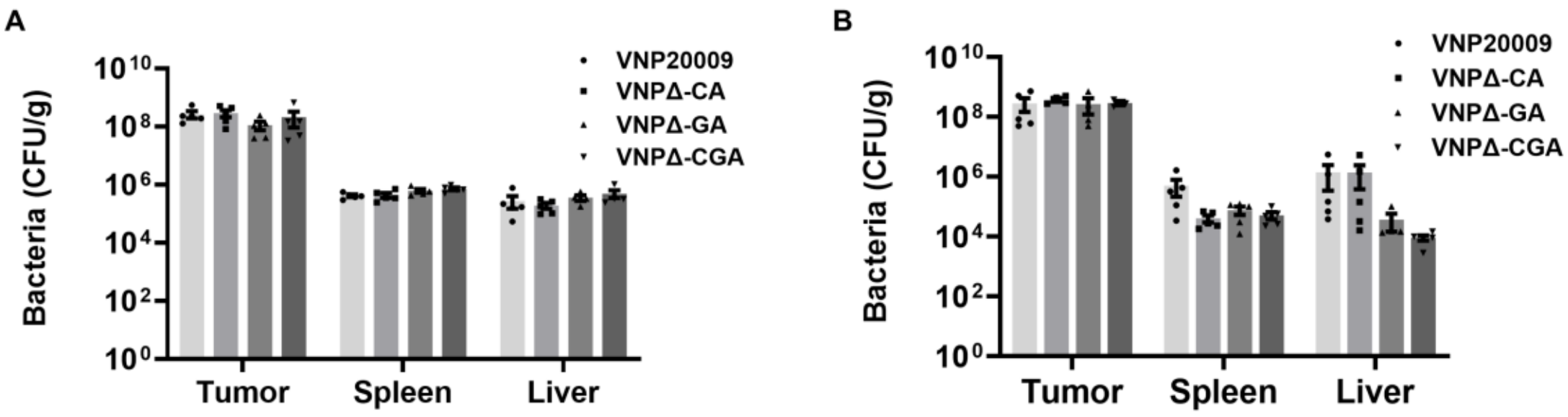

Figure S7. Quantification of bacterial colonization in tumors and healthy organs (spleen, liver) of CT26 tumor-bearing mice.

(A) Bacterial colonization at day 3 after bacterial administration (mean ± SEM, *n*=5).

(B) Bacterial colonization at the end of therapy, i.e., on day 12 after the initial administration (mean ± SEM, *n*=5).

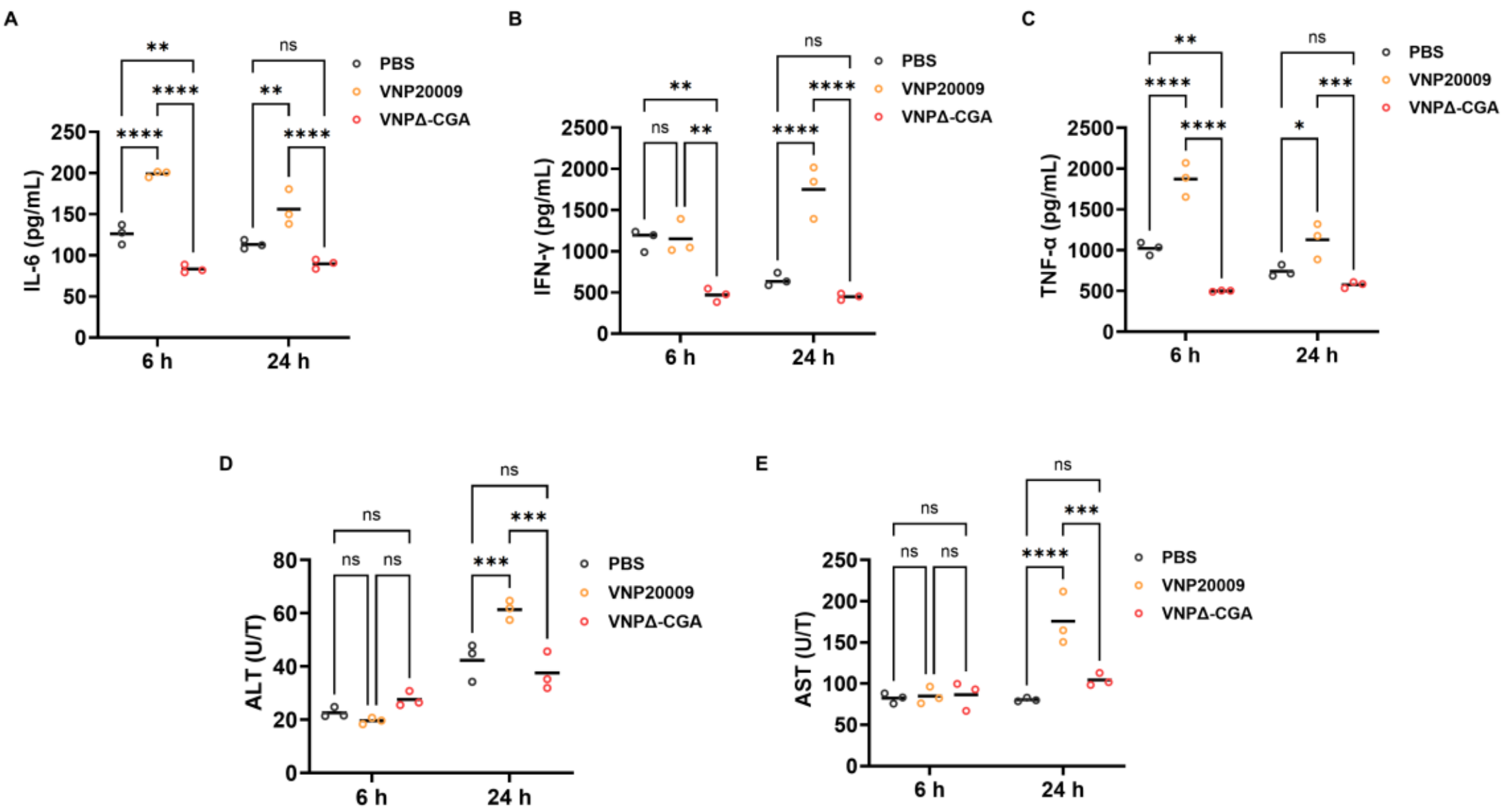

Figure S8. Evaluation of acute systemic inflammatory response and hepatic safety after intravenous administration of bacteria.

(A-C) Serum levels of pro-inflammatory cytokines, including IL-6, IFN-γ, and TNF-α, in mice after intravenous administration of PBS, VNP20009, or VNPΔ-CGA at 6 h and 24 h post-injection. (mean ± SEM, n = 3). P values were analyzed by two-way ANOVA followed by Tukey's multiple comparisons test. ns, $p > 0.05$, * $p \leq 0.05$, *** $p \leq 0.001$, **** $p \leq 0.0001$.

(D and E) Serum ALT and AST levels in mice after intravenous administration of different treatments at 6 h and 24 h post-injection. (mean ± SEM, n = 3). P values were analyzed by two-way ANOVA followed by Tukey's multiple comparisons test. ns, $p > 0.05$, *** $p \leq 0.001$, **** $p \leq 0.0001$.

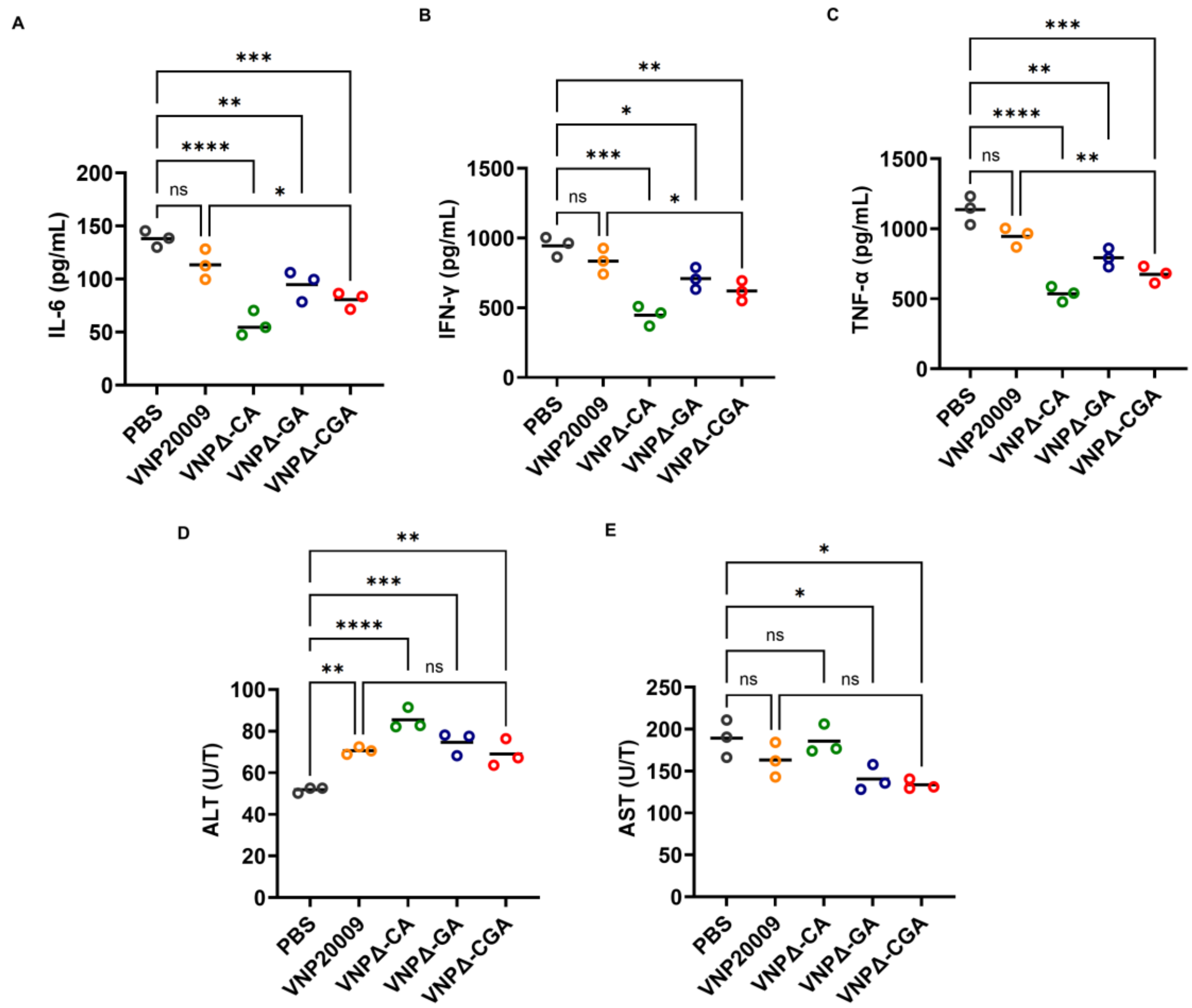

Figure S9. Evaluation of systemic inflammatory response and liver function after bacterial treatment.

(A-C) Serum levels of pro-inflammatory cytokines, including IL-6, IFN-γ, and TNF-α, in CT26 tumor-bearing mice after different treatments (mean ± SEM, *n*=3). *P* values were analyzed by one-way ANOVA, ns $p > 0.05$, *$p \leq 0.05$, **$p \leq 0.01$, ***$p \leq 0.001$, ****$p \leq 0.0001$.

(D and E) Serum ALT and AST levels in CT26 tumor-bearing mice after different treatments. (mean ± SEM, *n*=3). *P* values were analyzed by one-way ANOVA, ns $p > 0.05$, *$p \leq 0.05$, **$p \leq 0.01$, ***$p \leq 0.001$, ****$p \leq 0.0001$.

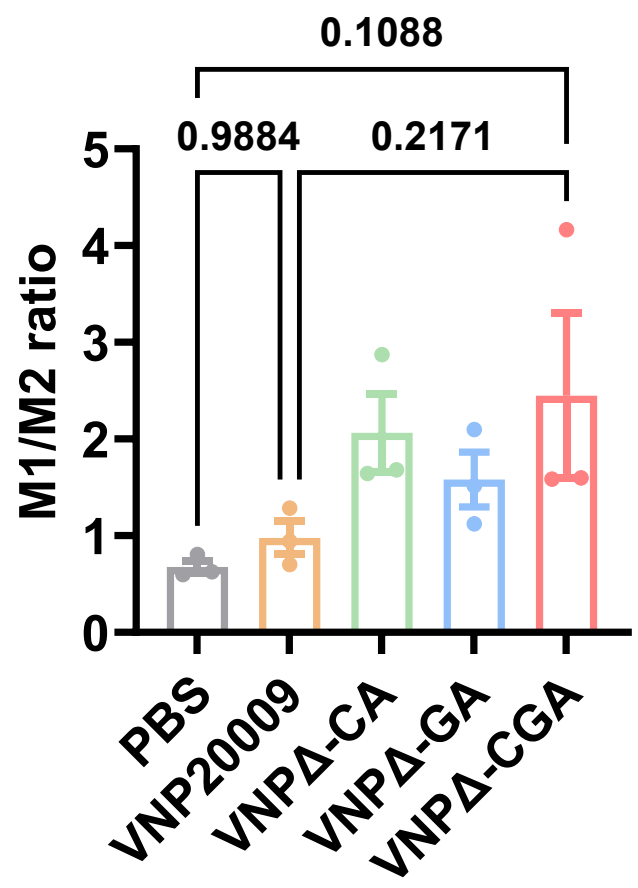

Figure S10. Quantification of the ratio of M1-like to M2-like macrophages in different treatment groups (mean ± SEM, *n*=3). *P* values were analyzed by one-way ANOVA.

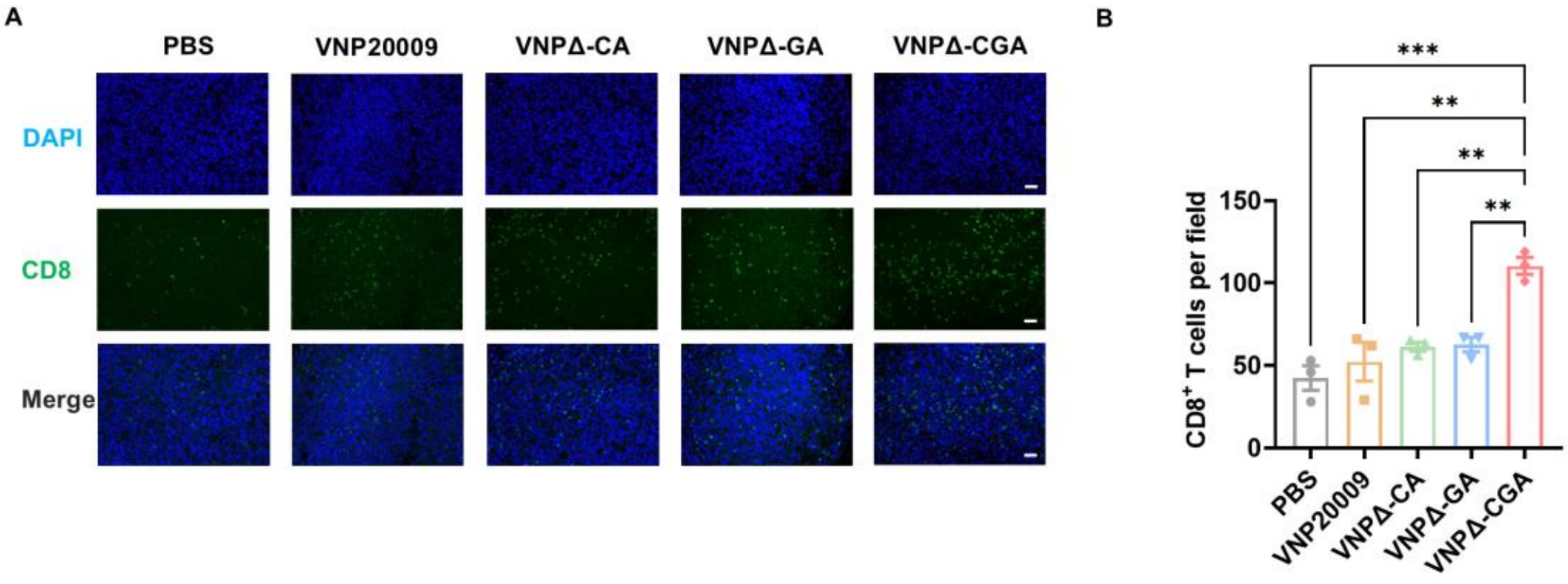

Figure S11. Immunofluorescence analysis of $CD8^+$ T cells in tumor tissues.

(A) Representative immunofluorescence images of tumor section from different treatment groups (green:$CD8^+$ T cells, blue: nuclei, scale bar: 100 μm).

(B) Quantification of $CD8^+$ T cells per field in different treatment groups using ImageJ (mean ± SEM, *n*=3). *P* values were analyzed by one-way ANOVA, $^{**}p \le 0.01$, $^{***}p \le 0.001$.

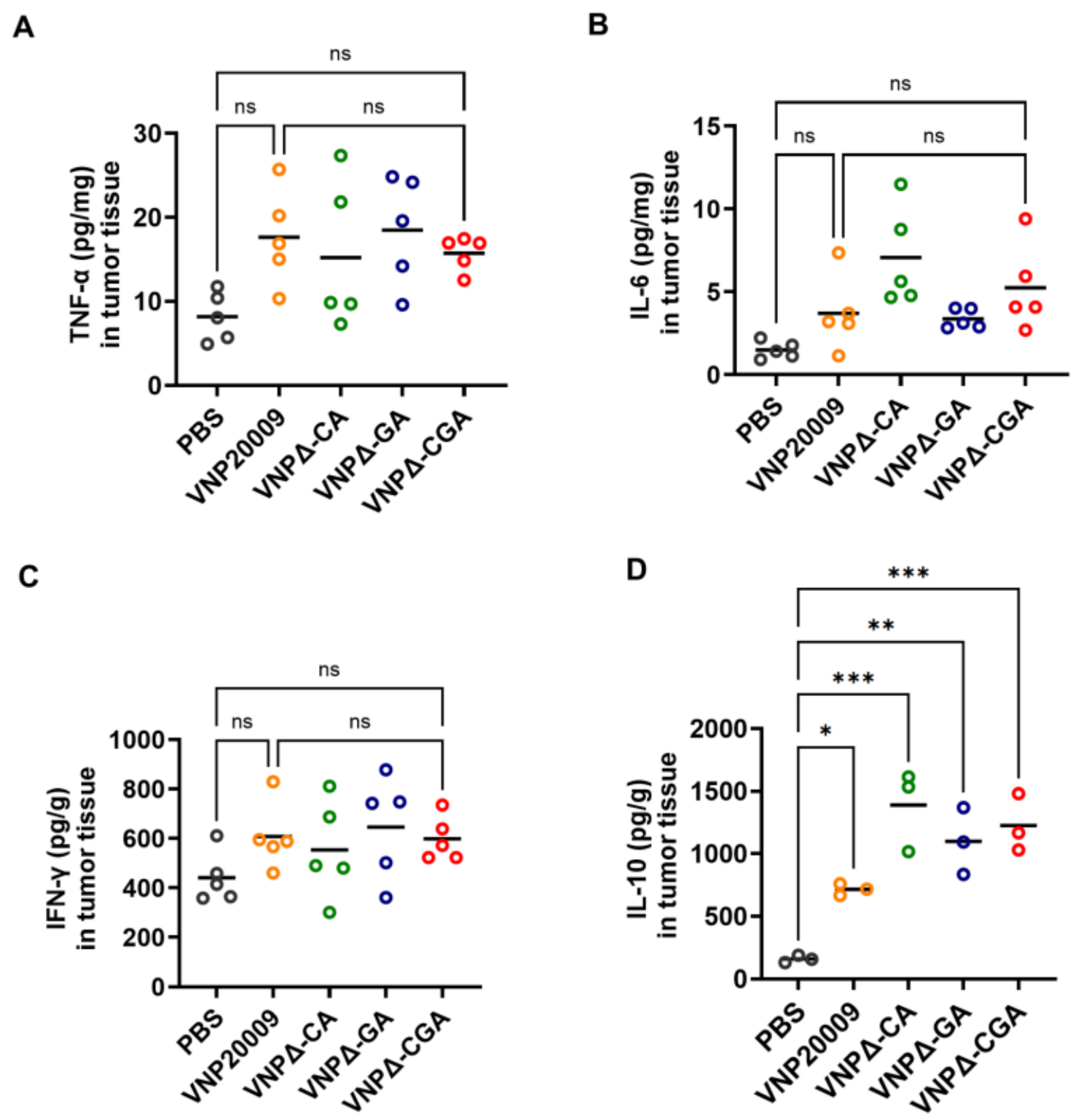

Figure S12. Intratumoral cytokine levels after bacterial treatment.

(A-D) Levels of TNF-α, IL-6, IFN-γ, and IL-10 in CT26 tumor tissues from different treatment groups (mean ± SEM, *n*=5). *P* values were analyzed by one-way ANOVA, ns $p > 0.05$, *$p \leq 0.05$, **$p \leq 0.01$, ***$p \leq 0.001$.

Table S1: Signal peptides and solubility tag applied and their amino acid sequences.

Signal sequences	Amino acid sequences	Solubility tag	Amino acid sequence
OmpC	MMKRKILAAVIPALLAAATANA	InfB	TDVTLKA
OmpF	MKVKVLSLLVPALLVAGAANA		

Table S2: Strains utilized and constructed in this study

Strains	Characteristics	CD47nb module	Peptide for CD47nb secretion	GM-CSF module	Peptide for GM-CSF secretion	Balanced plasmid lethal system	LuxCDABE module
Salmonella typhimurium VNP20009	unengineered	-	-	-	-	-	-
VNPΔ*asd*	*asd*$^-$	-	-	-	-	-	-
VNP-C	CD47nb	+	OmpA	-	-	-	-
VNP-MIS-G	GM-CSF	-	-	+	MISSSIS	-	-
VNP-CG	CD47nb, GM-CSF	+	OmpA	+	OmpF-infB	-	-
VNPΔ-CA	CD47nb, *asd*	+	OmpA	-	-	+	-
VNPΔ-GA	GM-CSF, *asd*	-	-	+	OmpF-infB	+	-
VNPΔ-CGA	CD47nb, GM-CSF, *asd*	+	OmpA	+	OmpF-infB	+	-
VNP-CG-Lux	CD47nb, GM-CSF, LuxCDABE	+	OmpA	+	OmpF-infB	-	+
VNPΔ-CGA-Lux	CD47nb, GM-CSF, *asd* LuxCDABE	+	OmpA	+	OmpF-infB	+	+

Table S3: Some primers and gene sequences used in this study

Name	Sequences
H-pWT g-R	tgatgctcgatgagtttttctaaacgccatgggcattaaaaaatgc
kana-F	ttagaaaaactcatcgagcatcaaatgaaactg
H-kana-R	aataatattgaaaaaggaagagtatgagccatattcaacgggaaacg
pWT g-F	actcttcctttttcaatattattgaagcatttatcagg
luxR-R	gagggtcattatatttgctgaggagtg
Plux-F	ggtacctttctcctctttaatgaattcattcg
H-CD47nb-R	tcattaaagaggagaaaggtaccatgaagaaaaccgcgattgcg
CD47nb-F	tcagccaccggtttccgg
H-luxI-R	gtctgccggaaaccggtggctgagaattcattaaagaggagaaaggtaccatgac
luxI-F	ttaatttaagactgctttttttaaactgttcattaataggcatag
H-Ter-R	ttaaaaaagcagtcttaaattaaacgcgtgctagaggcatc
H-asd-F	cagttcgctgcgctcacggctctctacgccaactggcgc
guC-R	agagccgtgagcgcag
asd-R	atgaaaaatgttggttttatcggctgg
H-0035-F	ccgataaaaccaacatttttcatctagtattctcctctttaatctctagagctagc
GM CSF-R	caagcgtaatctggaacgtcatatgg
H-yfiD gu-F	tatgacgttccagattacgcttgatctagaccaggcatcaaataaaacgaaag
H-yfid-R	ctgaatattccttttgtctccacccgcataagtttatgtaaaaaaataaaatccatttg
gu-R	gtggagacaaaaggaatattcagcgatttgc
H-cmr-F1	agcattcgacgcagcggctcggcggcgccccataacaactggtttacgccccgccctgc
H-cmr-F2	ctacgccaactggcgcagcattcgacgcagcg
H-cmr-R1	cgcggaatggtcggctctgttctcatgcaacgtcgatatctggcgaaaatgagacgttg
H-cmr-R2	atgaaaaatgttggttttatcggctggcgcggaatggtcggc
OmpA-flag-CD47nb	tcagccaccggtttccggcagaccgccgctactcacggtcacctgggtgccctgaccccaatagcgacgccattctgtaccatcgcga<br>tcggtgctaataactgcggtgcaataataaactgcggtatcttccggtttcaggctattcatctgcaggaaaacgctatttttttgcatcacgg<br>ctaatggtgaaacgatctttaacactatcgcgataaatggcttcatcgccaccggtgcttgctgccacccattcacggcgtttacccgggg<br>cctgtctgtaccagcccatgtcgttgattttaaaaataatgccacttgcggcacaactcagacgcagactaccacccggttccaccaggc

	caccaccgctttcaaccagctgaacctgtgccttgtcatcgtcgtccttgtagtccgcttgcgcaacggtcgcaaaacccgccagcgcc<br>accgcaatcgcaatcgcggttttcttcat
OmpF-infB-GM-CSF-HA	atgatgaagcgcaaaatcctggcagcggtgatccctgccctgctggctgctgcaaccgcaaacgcaacagatgtaaccctaaaagcg<br>gcacccacccgctcacccatcactgtcacccggccttggaagcatgtagaggccatcaaagaagccctgaacctcctggatgacatgc<br>ctgtcacgttgaatgaagaggtagaagtcgtctctaacgagttctccttcaagaagctaacatgtgtgcagacccgcctgaagatattcga<br>gcagggtctacggggcaatttcaccaaactcaagggcgccttgaacatgacagccagctactaccagacatactgcccccccaactccg<br>gaaacggactgtgaaacacaagttaccacctatgcggatttcatagacagccttaaaacctttctgactgatatcccctttgaatgcaaaa<br>aaccaggccaaaaatacccatacgatgttcctgactatgcgggctatccgtatgacgtcccggactatgcaggatcctatccatatgacg<br>ttccagattacgcttga
yfiD-m	acccgcataagtttatgtaaaaaaataaaatccatttgcgaccatacttgatgtgtggtttttattgatttaaatcaaagattcaagggtgtttg<br>aggagtatatatacactcaagcaacaatggtttt
luxR	atgatatataacacgcaaaacttgcgacaaacaataggtaaggataaagagatgggtatgaaaaacataaatgccgacgacacataca<br>gaataattaataaaattaaagcttgtagaagcaataatgatattaatcaatgcttatctgatatgactaaaatggtacattgtgaatattatttac<br>tcgcgatcatttatcctcattctatggttaaatctgatatttcaattctagataattaccctaaaaaatggaggcaatattatgatgacgctaattt<br>aataaaatatgatcctatagtagattattctaactccaatcattcaccaattaattggaatatatttgaaaacaatgctgtaaataaaaaatctc<br>caaatgtaattaaagaagcgaaaacatcaggtcttatcactgggtttagtttccctattcatacggctaacaatggcttcggaatgcttagttt<br>tgcacattcagaaaaagacaactatatagatagtttatttttacatgcgtgtatgaacataccattaattgttccttctctagttgataattatcga<br>aaaataaatatagcaaataataaatcaaacaacgatttaaccaaaagagaaaaagaatgtttagcgtgggcatgcgaaggaaaaagctc<br>ttgggatatttcaaaaatattaggctgcagtgagcgtactgtcactttccatttaaccaatgcgcaaatgaaactcaatacaacaaaccgct<br>gccaaagtatttctaaagcaattttaacaggagcaattgattgcccatactttaaaaattaa
luxI	ttaatttaagactgctttttaaactgttcattaataggcatagacaatacaaccgatttagtatcacctaatacatgaatttctttgtctccaata<br>cgatgacaaggaactttaatacgctttaaaaatcgctctattgctgttgatgttactgttacatattctgtaataccttgactaacagcgtgttta<br>tatatagcttcaaatagtttcattgtaatttcactagcagagttatttatctttgagctatttttacctacagcaaaacgacttaattcgactatatt<br>aggatctttgggagcactctgttgaccaagcaattcaggaaaaacacttttcagcatataatcacctgttgtaggtaataaacgccagcat<br>ccacttacattttcagtatcatcacaagcataaatatattctgcatttgagttatcatactcatctgattcaaggttattttctacaactaagtccc<br>actcaagtctttgcttaaacacttgataacgaagacttagaatacctttatactcctccgatggaattgccaaaaaatccgatttttttatcatta<br>tagtcat
asd	ctacgccaactggcgcagcattcgacgcagcggctcggcggcgccccataacaactggtcgcctacggtaaacgccgacaagaact<br>ctggccccatgttcagcttacgcagacgaccaaccggcgtagtcaacgtgccggtcaccgccgccggggttaattcgcgcatagtgat

atcacgatcgttcggcaccactttcgcccacggattatgtgccgccagcagttcttccaccgtcggaatggatacctctttttcagcttgat

ggtgaacgcctggctgtgacagcgcagcgcgccgacgcgcacacacaaaccatcaaccggaatcacagaggcagtattgagaatct

tgttggtttccgcctggcctttccactcttcgcggctctggccgttatcgagctgtttgtcgatccaggggatcaggcttcccgccagcggt

acgccaaagttatcaaccggcagctcgccgctgcgggtcaatgccgtaactttgcgttcaatatcaagaattgcggaagacggcgtcg

ccagttcatcggcgacatggccatacaactgacccatctgggttaacagctcgcgcatatggcgcgcgccgccgccggaggcggcct

gataggtcgcgacggatacccagtcaacgagattatgggcaaagagaccgcccagcgacatcaacatcaggctaacggtacagttac

cgcccacaaaggtcttcacgccattgttcaggccgtcggtaatcacgtcctggttgaccgggtcgagaataataatggcatcatctttcat

gcgcagcgtagaagccgcatcaatccagtaaccctgccatccgctttcgcgcagctttggataaatttcgttggtataatcgccgccctg

gcaggtcacgatgatatcgagcgcttttagcgcatccagatcaaaagcgtcctgtagcgtgccggtggaggtgtcgccgaaggtgggc

gccgcctgtccaaactgggaggtagaaaagaaaacagggcgaatagcgtcgaaatcgcgctcctctaccatgcgttgcatgagaaca

gagccgaccattccgcgccagccgataaaaccaacatttttcat
