# Supplementary material for: In situ-activated engineered bacteria release combined dual-therapeutic proteins for tumor immunotherapy
Source: iScience. 2026 Nov 2;29(11):118090. doi: 10.1016/j.isci.2026.118090 (PMC13755573; doi:10.1016/j.isci.2026.118090)
Supplement: Supplementary file 2 [file mmc2.pdf]

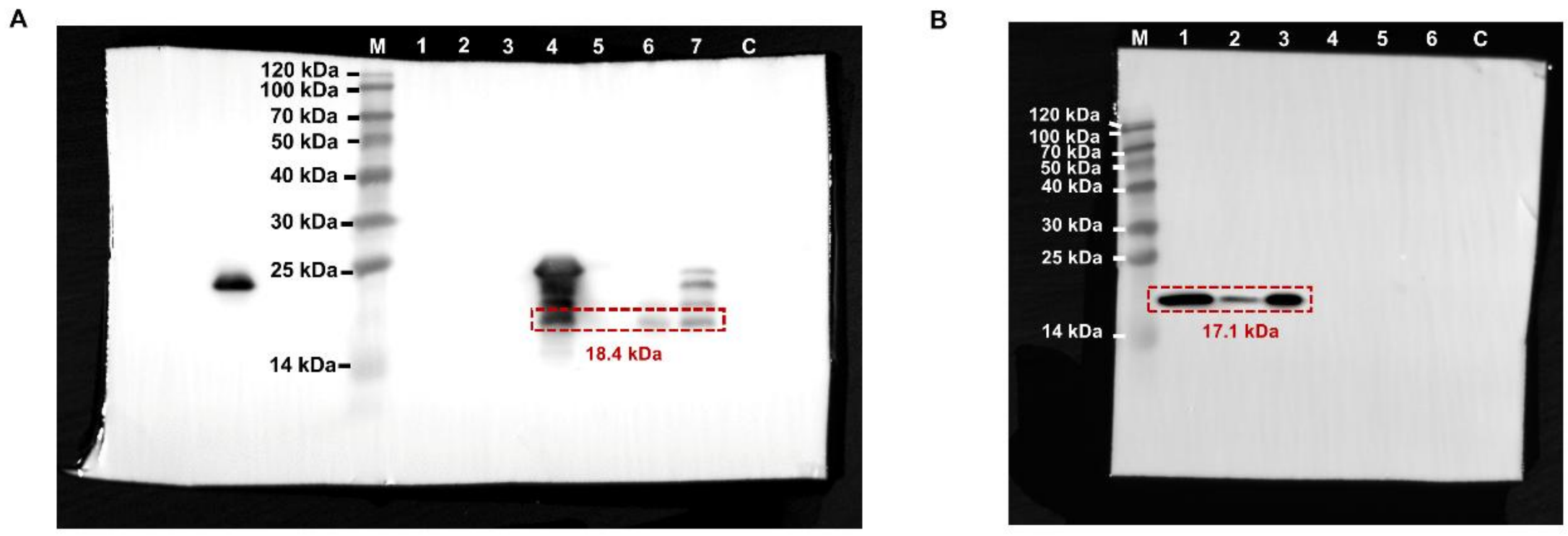

Data S1. Unprocessed Western blot images of Figures 1G and 1I.

(A) The unprocessed Western blot image of Figure 1G. M, marker; lanes 1-7, experimental samples; C, loading control.

(B) The unprocessed Western blot image of Figure 1I. M, marker; lanes 1-6, experimental samples; C, loading control.

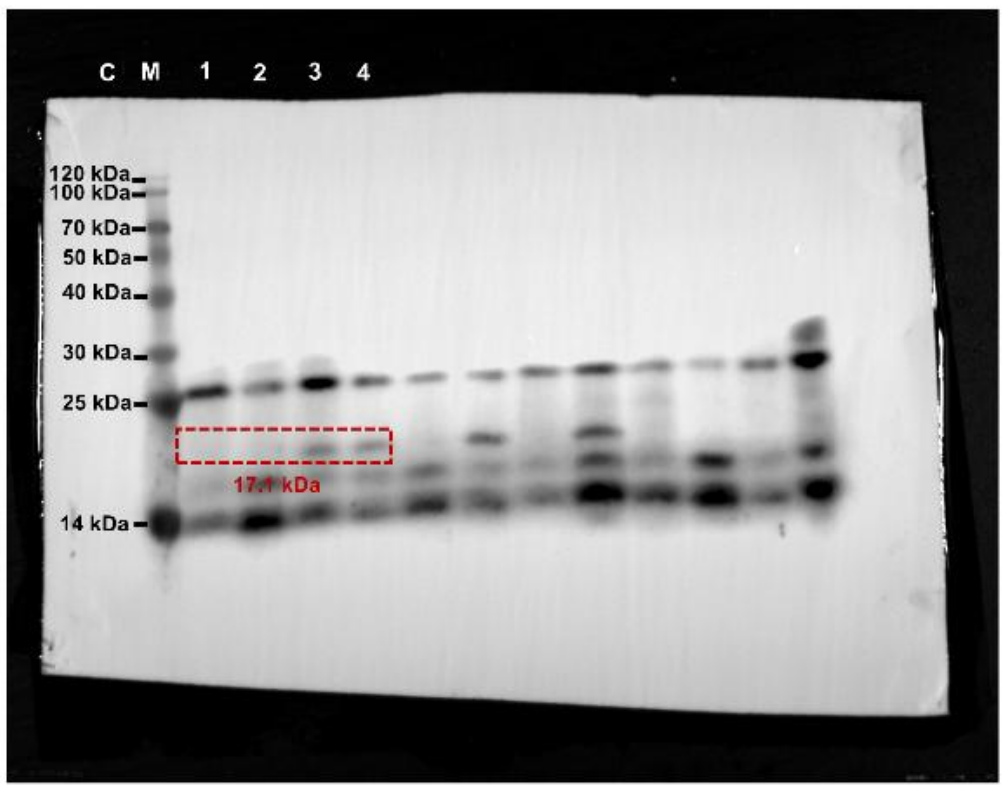

Data S2. The unprocessed WB image of Figure 4B. M, marker; lanes 1-4, experimental samples; C, loading control.
